# Recently evolved combination of unique sulfatase and amidase genes enables bacterial degradation of the wastewater micropollutant acesulfame worldwide

**DOI:** 10.3389/fmicb.2023.1223838

**Published:** 2023-07-27

**Authors:** Maria L. Bonatelli, Thore Rohwerder, Denny Popp, Yu Liu, Caglar Akay, Carolyn Schultz, Kuan-Po Liao, Chang Ding, Thorsten Reemtsma, Lorenz Adrian, Sabine Kleinsteuber

**Affiliations:** ^1^Department of Environmental Microbiology, Helmholtz Centre for Environmental Research—UFZ, Leipzig, Germany; ^2^Department of Environmental Biotechnology, Helmholtz Centre for Environmental Research—UFZ, Leipzig, Germany; ^3^Department of Analytical Chemistry, Helmholtz Centre for Environmental Research—UFZ, Leipzig, Germany; ^4^Institute of Analytical Chemistry, University of Leipzig, Leipzig, Germany; ^5^Chair for Geobiotechnology, Technische Universität Berlin, Berlin, Germany

**Keywords:** pathway evolution, treatment wetland, sulfamic acid derivative, mobile genetic element, horizontal gene transfer

## Abstract

Xenobiotics often challenge the principle of microbial infallibility. One example is acesulfame introduced in the 1980s as zero-calorie sweetener, which was recalcitrant in wastewater treatment plants until the early 2010s. Then, efficient removal has been reported with increasing frequency. By studying acesulfame metabolism in alphaproteobacterial degraders of the genera *Bosea* and *Chelatococcus*, we experimentally confirmed the previously postulated route of two subsequent hydrolysis steps via acetoacetamide-N-sulfonate (ANSA) to acetoacetate and sulfamate. Genome comparison of wildtype *Bosea* sp. 100-5 and an acesulfame degradation-defective mutant revealed the involvement of two plasmid-borne gene clusters. The acesulfame-hydrolyzing sulfatase is strictly manganese-dependent and belongs to the metallo beta-lactamase family. In all degraders analyzed, it is encoded on a highly conserved gene cluster embedded in a composite transposon. The ANSA amidase, on the other hand, is an amidase signature domain enzyme encoded in another gene cluster showing variable length among degrading strains. Transposition of the sulfatase gene cluster between chromosome and plasmid explains how the two catabolic gene clusters recently combined for the degradation of acesulfame. Searching available genomes and metagenomes for the two hydrolases and associated genes indicates that the acesulfame plasmid evolved and spread worldwide in short time. While the sulfatase is unprecedented and unique for acesulfame degraders, the amidase occurs in different genetic environments and likely evolved for the degradation of other substrates. Evolution of the acesulfame degradation pathway might have been supported by the presence of structurally related natural and anthropogenic compounds, such as aminoacyl sulfamate ribonucleotide or sulfonamide antibiotics.

## 1. Introduction

Acesulfame potassium (ACE-K) is an artificial sweetener used in food and beverages, pharmaceuticals and personal care products. It was discovered in the late 1960s (Clauß and Harald, 1973) and is a zero-calorie, high-potency sweetener that is 200 times sweeter than sucrose, with good storage and temperature stabilities (Klug and von Rymon Lipinski, 2012). It received its first approval of use in the UK in 1983 and has been used worldwide since the 1990s (Klug and von Rymon Lipinski, 2012). After human consumption, the acesulfame anion (ACE) is readily absorbed but not metabolized, and eventually excreted unchanged via the urinary tract. Consequently, ACE is present in domestic wastewater in a range between 10 and 100 μg L^−1^ (Lange et al., 2012) and can reach other aquatic environments (Buerge et al., 2009). Due to its hydrophilicity and persistence, ACE became an ideal anthropogenic wastewater tracer (Lange et al., 2012). Recalcitrance of ACE in wastewater treatment plants (WWTPs) was reported first in 2009 (Buerge et al., 2009), and its persistence in surface waters, groundwater and even tap water was observed in the USA (Subedi and Kannan, 2014; Schaider et al., 2016), Canada (Liu et al., 2014), China (Gan et al., 2013), Germany (Nodler et al., 2013) and other countries in the early 2010s. However, reports on ACE biodegradation in WWTPs started to appear in the later 2010s. Two studies conducted in Australia (Cardenas et al., 2016) and China (Yang et al., 2017) revealed high percentages (92% and 85%, respectively) of ACE removal in WWTPs. When analyzing 13 WWTPs in Switzerland and Germany, Castronovo et al. (2017) noticed 57 to 97% ACE removal and identified sulfamic acid/the sulfamate ion as the transformation product. Likewise, Kahl et al. (2018) reported biodegradation of >85% in nine WWTPs in Germany and observed a seasonality of ACE removal with highest efficiency in summer and autumn. These results challenged the perception of ACE recalcitrance and clearly indicated biodegradation as the removal mechanism.

**Figure 1:**
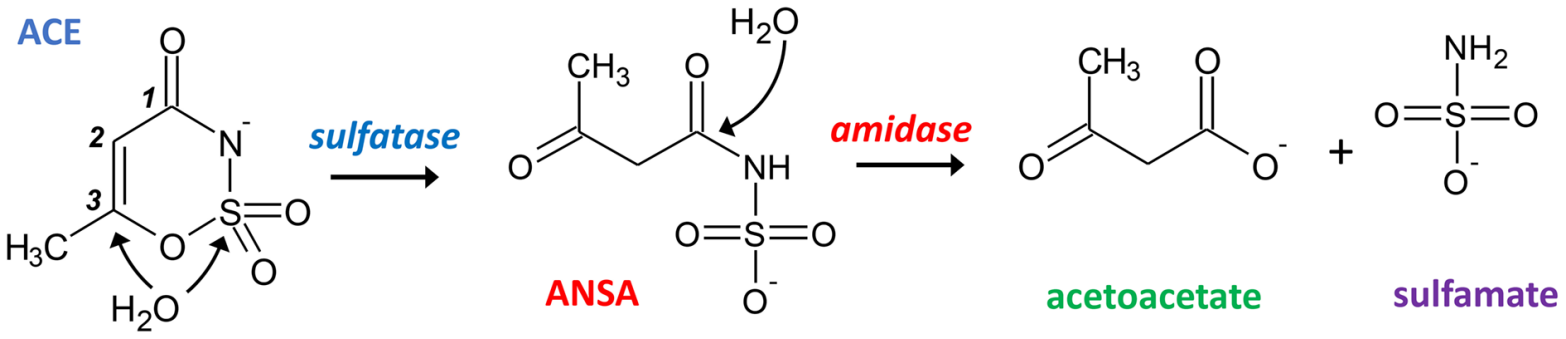
Proposed hydrolytic pathway for bacterial degradation of ACE. Initially, a sulfatase catalyzes the attack of either the sulfur or C3 atom of ACE resulting in the formation of ANSA. In a subsequent step, an amidase hydrolyzes the linear amide intermediate to acetoacetate and sulfamate.

The reports on emerging ACE biodegradability in WWTPs inspired research on the microorganisms and metabolic pathways involved with this process. In a first study of our lab (Kahl et al., 2018), ACE-degrading bacteria were enriched from sludge sampled from a treatment wetland in 2015 (located in Mockrehna, Germany). We used mineral medium with 125 μg L^−1^ to 125 mg L^−1^ ACE-K as sole carbon source and showed evidence for its complete mineralization. From the resulting community, which was dominated by *Alpha-and Gammaproteobacteria* (Kahl et al., 2018), we isolated a pure culture identified as *Bosea* sp. 3-1B (Kleinsteuber et al., 2019). Three more strains (*Bosea* sp. 100-5, *Chelatococcus* sp. 1 g-2 and 1 g-11) were isolated later using fresh samples from the same wetland by direct enrichment on 0.1 to 1 g L^−1^ ACE-K (Kleinsteuber et al., 2019). The latter three strains completely degraded 1 g L^−1^ ACE-K within 8 to 9 days and formed sulfamate in stoichiometric amounts during batch cultivation. Additionally, acetoacetamide-N-sulfonate (ANSA) was detected transiently in culture supernatants, confirming the assumption of Castronovo et al. (2017) who suggested ANSA as an intermediate of ACE degradation. Based on this finding, we proposed a catabolic pathway of two subsequent hydrolysis steps via ANSA to acetoacetate as shown in Figure 1 (Kleinsteuber et al., 2019).

Another group investigated ACE-degrading communities enriched from activated sludge (sampled at a WWTP located in Hong Kong, China) and found that the most abundant metagenome-assembled genomes (MAGs) belonged to *Planctomycetes*, *Alpha- and Gammaproteobacteria* (Huang et al., 2021). These authors also isolated two ACE-mineralizing *Chelatococcus* strains (YT9 and HY11) and compared their genomes with those of two *Chelatococcus* type strains that do not degrade ACE. They found a total of 812 protein-coding genes that were present in both ACE-degrading strains but not in *Chelatococcus* type strains DSM 6462 and DSM 101465. However, they could not further narrow down the ACE degradation genes. Recently, the group of bacterial genera with representatives capable of ACE mineralization was extended by *Shinella*, and based on comparative genomics it was speculated that the degradation is a plasmid-mediated process (Huang et al., 2022).

Despite these advances in isolating ACE-degrading pure cultures and sequencing the genomes of a few strains, the ACE-degrading enzymes have not yet been identified, and the proposed pathway still needs to be confirmed. Hence, the genetic background of this recently emerged catabolic trait and its evolutionary origin remained elusive. The present study aimed to elucidate the ACE metabolism in our previously isolated strains (Kleinsteuber et al., 2019) and some other ACE degraders that we have isolated from WWTPs located in Germany. Based on comparative genomics, heterologous expression and biochemical analysis, we identified the genes responsible for ACE degradation and surveyed their distribution in public sequence databases. Additionally, we discuss the mechanisms that might have established this pathway by combination of preexisting catabolic genes and facilitated its fast distribution by horizontal gene transfer.

## 2. Materials and methods

### 2.1. Isolation and cultivation of bacterial strains

*Bosea* sp. 3-1B was previously isolated from an ACE-degrading enrichment culture (Kahl et al., 2018; inoculum sludge sampled in July 2015, see also Table 1). *Bosea* sp. 100-5, *Chelatococcus* sp. 1 g-2 and *Chelatococcus* sp. 1 g-11 originated from the same treatment wetland sampled in February 2019. The strains were isolated by plating on R2A agar (Reasoner and Geldreich, 1985) after enrichment in DSMZ 462 mineral medium (see Supplementary material for recipe) with 0.5 or 5 mM ACE as sole carbon source (Kleinsteuber et al., 2019). *Chelatococcus asaccharovorans* WSA4-1, *Chelatococcus* sp. WSC3-1, *Chelatococcus asaccharovorans* WSD1-1, *Chelatococcus* sp. WSG2-a and *Shinella* sp. WSC3-e were isolated from enrichment cultures in DSMZ 461 mineral medium (see Supplementary material for recipe) with 5 mM ACE inoculated with activated sludge from four WWTPs in Germany (sampled in 2020, see Table 1). The strains were identified by Sanger sequencing of 16S rRNA genes (Lane, 1991) and maintained on DSMZ 461 agar with 5 mM ACE incubated at 30°C. Liquid cultures to study growth and degradation kinetics or for enzyme assays and DNA extraction were set up in DSMZ 461 or DSMZ 462 medium with the respective carbon source (2.5 to 10 mM ACE, 10 mM 3-hydroxybutyrate or 10 mM succinate; supplied by Sigma-Aldrich as potassium or sodium salts). Inability of *Paraburkholderia sartisoli* LMG 24000 (obtained as DSM 23088 from DSMZ, Braunschweig, Germany) to degrade ACE was tested at 30°C during growth on DSMZ 461 with 3-hydroxybutyrate (2.5 mM ACE not degraded) and by incubation on DSMZ 461 with 5 mM ACE as only substrate (no growth observed).

**Table 1:** Information on the bacterial genomes analyzed in this study.

Strain	Sampling site/isolation origin	Sampling date	Localization of ACE clusters 1 and 2	Size of contigs with ACE clusters (bp)	Assembly accession	References
*Bosea* sp. 100-5	Treatment wetland Langenreichenbach, Mockrehna, Germany (51°30′N 12°53′E)	2019	Contig 4	40,931	GCA_930633465	Kleinsteuber et al. (2019)
*Bosea* sp. 100-5 Mut1	Spontaneous mutant of *Bosea* sp. 100-5	Isolated in 2021	Contig 56 (only cluster 2)	9,696 (only cluster 2)	GCA_946047605	This work
*Bosea* sp. 3-1B	Treatment wetland Langenreichenbach, Mockrehna, Germany (51°30′N 12°53′E)	2015	Contig 3	43,141	GCA_930633495	Kleinsteuber et al. (2019)
*Chelatococcus* sp. 1 g-2	Treatment wetland Langenreichenbach, Mockrehna, Germany (51°30′N 12°53′E)	2019	Contig 3	41,507	GCA_930633515	Kleinsteuber et al. (2019)
*Chelatococcus* sp. 1 g-11	Treatment wetland Langenreichenbach, Mockrehna, Germany (51°30′N 12°53′E)	2019	Contig 6	45,665	GCA_930633505	Kleinsteuber et al. (2019)
*Chelatococcus asaccharovorans* WSA4-1	WWTP Markranstädt, Germany (51°18′N 12°12′E)	2020	Contig 5	45,942	GCA_930633525	This work
*Chelatococcus* sp. WSC3-1	WWTP Rosental, Leipzig, Germany (51°21′N 12°20′E)	2020	Contig 2	45,505	GCA_930633455	This work
*Chelatococcus asaccharovorans* WSD1-1	WWTP Markkleeberg, Germany (51°17′N 12°21′E)	2020	Contig 6	41,507	GCA_930633485	This work
*Chelatococcus* sp. WSG2-a	WWTP Wiedemar, Germany (51°28′N 12°11′E)	2020	Contig 1	45,506	GCA_930633475	This work
*Chelatococcus* sp. YT9	WWTP Sha Tin, Hong Kong, China	2018	Contig 1 (cluster1) Contig 5 (cluster 2)	4,368,288 (cluster 1) 16,496 (cluster 2)	GCA_018398315	Huang et al. (2021)
*Chelatococcus* sp. HY11	WWTP Sha Tin, Hong Kong, China	2018	Contig 6 (only cluster 2)	18,060 (only cluster 2)	GCA_018398335	Huang et al. (2021)
*Shinella* sp. WSC3-e	WWTP Rosental, Leipzig, Germany (51°21′N 12°20′E)	2020	Contig 2 (only cluster 2)	41,586 (only cluster 2)	GCA_945994535	This work
*Shinella* sp. HY16	WWTP Sha Tin, Hong Kong, China (22.17 N 114.08 E)	2021	Contig 11 (only cluster 2)	41,603 (only cluster 2)	GCA_028534175	Huang et al. (2022)
*Shinella* sp. YE25	WWTP Sha Tin, Hong Kong, China (22.17 N 114.08 E)	2021	Contig 13 (only cluster 2)	41,586 (only cluster 2)	GCA_028534295	Huang et al. (2022)
*Shinella* sp. YZ44	WWTP Sha Tin, Hong Kong, China (22.17 N 114.08 E)	2021	Contig 11 (only cluster 2)	41,603 (only cluster 2)	GCA_028534455	Huang et al. (2022)

To select an ACE degradation-defective mutant, *Bosea* sp. 100-5 was grown in R2A medium for 12 transfers and plated on R2A agar in dilution series to obtain single colonies. Isolates were screened for growth in liquid culture on either ACE or succinate as sole carbon source. One isolate that grew on succinate but not on ACE was identified and designated as *Bosea* sp. 100-5 Mut1.

### 2.2. Genome sequencing

Genomic DNA was extracted from ACE-grown cells (DSMZ medium 461 with 5 mM ACE) with the NucleoSpin Microbial DNA kit (Macherey-Nagel, Düren, Germany) according to the manual and using wide-bore tips. DNA concentration was measured with the Qubit dsDNA BR Assay Kit (Thermo Fisher Scientific, Rockford, IL, United States); DNA integrity was checked by gel electrophoresis.

Genomes of all strains except *Bosea* sp. 100-5 Mut1 were sequenced using a combination of long-read and short-read approaches. Long-read sequencing was done on the MinION MK1b platform (Oxford Nanopore Technologies, Oxford, United Kingdom) as previously described (Liu et al., 2020) using Guppy v4.0.15 for basecalling. Nanopore read data was downsampled per isolate to about 150x coverage using filtlong v0.2.0^1^ with a priority on read length (—length_weight 10). Short-read sequencing of strain 3-1B was conducted on the Illumina MiSeq platform (Illumina, San Diego, CA, United States) using the NEBNext Ultra II FS DNA library prep kit (New England Biolabs, Frankfurt, Germany) to prepare a 2 × 300 bp library. Short-read sequencing of the other strains was performed on the Illumina NovaSeq platform (2 × 150 bp; Genewiz, Leipzig, Germany). Short and long reads were assembled with a hybrid approach using Unicycler v0.4.9 (Wick et al., 2017) with default parameters.

Genome sequencing of *Bosea* sp. 100-5 Mut1 was conducted on the Illumina NextSeq 2000 platform (2 × 150 bp; StarSeq, Mainz, Germany). After assembly with SPAdes version 3.15.2 (Prjibelski et al., 2020), contigs smaller than 500 bp were excluded from the assembly. Platanus v1.2.1 (Kajitani et al., 2014) was used for scaffolding and Mauve 20150226 (Darling et al., 2004) for reordering the contigs using the wildtype genome of *Bosea* sp. 100-5 as reference.

### 2.3. Genome annotation and comparative genomics

The MicroScope pipeline was used for the prediction and functional annotation of coding sequences (CDS; Vallenet et al., 2020). The tools GTDB-tk 1.1.2 (Chaumeil et al., 2019) and CheckM 1.3.0 (Parks et al., 2015) were used for taxonomic classification and estimating genome completeness and contamination, respectively. Genome metrics is provided in Supplementary Table S1. To compare the genome of *Bosea* sp. 100-5 with *Bosea* sp. 100-5 Mut1, we used Geneious 10.0.9 (Kearse et al., 2012), Mauve and Proksee 1.1.3 (Stothard and Wishart, 2005). Additionally, the Synteny Statistics tool of MicroScope was used to explore the similarity between the two genomes. Gene clusters of interest and surrounding genes were investigated in all genomes with cblaster version 1.3.11 (Gilchrist et al., 2021). Besides the genomes sequenced in this study, we included the genomes of five isolates and the MAG sequences of the corresponding BioProjects (PRJNA725625, PRJNA749893; Huang et al., 2021, 2022). Briefly, protein sequences of >100 amino acids were searched against a database built from genomes annotated with MicroScope applying default parameters, except for minimal nucleotide identity of 90%. For the genomes that did not present the gene clusters, tblastn was used to manually search for BOSEA1005_40015.

To look for genes related to BOSEA1005_40015 in public databases, we searched the NCBI and the JGI^2^ databases (both June 2023) using different strategies. In NCBI, we used tblastn with both nr and env_nr databases and with the whole-genome shotgun contigs (WGS) database. The tblastn search was limited to the following metagenomes: activated sludge (taxid:942017), aquatic (taxid:1169740), aquifer (taxid:1704045), bioreactor (taxid:1076179), bioreactor sludge (taxid:412754), drinking water (taxid:2651591), freshwater (taxid:449393), groundwater (taxid:717931), lagoon (taxid:1763544), lake water (taxid:1647806), pond (taxid:1851193), sludge (taxid:1592332) and wastewater (taxid:527639). In JGI, blastp search (E-value < 1 e-40) was done in the metagenome datasets wastewater treatment plant (365) and wastewater (439), and in the metatranscriptome datasets wastewater treatment plant (43) and wastewater (20). Metagenome dataset bioreactor (816) and metatranscriptome dataset bioreactor (259) were also investigated. For datasets presenting hits for the query sequence BOSEA1005_40015, we searched for other elements of the *Bosea* sp. 100-5 plasmid and used Geneious and Proksee to compare them. Predicted proteins with ambiguous annotation were searched for functional sites with the NCBI Conserved Domains tool (Lu et al., 2020) using CDD v3.20 and InterPro 93.0 (Finn et al., 2017).

### 2.4. Biochemical analyses

Crude extracts from bacterial cells were prepared in lysis buffer (10 mM Tris–HCl with 10% glycerol, pH 7.8) applying French press or disruption in a mixer mill with glass beads (Becher et al., 2018). Extracts were obtained by centrifugation at 20,000 x g and 4°C for 20 min. For removal of low molecular weight compounds interfering with HPLC analysis, protein extracts were purified with 10 kDa Amicon filters (Merck, Darmstadt, Germany). Protein concentration was determined with Pierce BCA Protein Assay Kit (Thermo Fisher Scientific) or Bradford reagent (AppliChem, Darmstadt, Germany) using bovine serum albumin as standard. For degradation assays and as analytical standards, ACE potassium salt (99% pure, Merck) and acetoacetate lithium salt (95% pure; abcr GmbH, Karlsruhe, Germany) were used. As ANSA is not commercially available, it was prepared employing the ACE-hydrolyzing activity of *Bosea* sp. 100-5. To separate ACE and ANSA hydrolase activities, size exclusion chromatography was used with protein crude extracts of ACE-grown cells, a Superdex 200 10/300 column (Cytiva, Marlborough, MA, United States) and 100 mM ammonium hydrogen carbonate buffer pH 7.82 as eluent. Fractions showing ACE hydrolytic activity were directly used to prepare up to 20 mM ANSA at 2-mL scale (for details see Supplementary material). The conversion was stoichiometric enabling calibration for ANSA (Supplementary Figure S1). ACE and ANSA hydrolysis activities were quantified in discontinuous HPLC-based assays at 30°C in lysis buffer. Samples were diluted in stop buffer (10 mM malonate, pH 4.0, 60°C) prior to HPLC analysis.

### 2.5. Heterologous expression of the hydrolase genes

Genes were synthesized and cloned into expression vector pET-28a(+)-TEV (BOSEA1005_40015 via *Bmt*I/*Bam*HI sites; BOSEA1005_40016 and BOSEA1005_40030 via *Nde*I/*Bam*HI sites; GenScript, Oxford, United Kingdom). Recombinant plasmids were transformed into *E. coli* Lemo21 (DE3; New England Biolabs). Resulting strains were grown at 30°C in lysogeny broth with 30 ppm chloramphenicol and 50 ppm kanamycin at 30°C until an optical density (600 nm) of 0.5. Then, 0.4 mM isopropyl β-d-1-thiogalactopyranoside were added and incubation proceeded at 17°C for 20 h (40016 and 40030) or 30°C for 4 h (40015). For expression of 40016 and 40030, cultures were supplemented with 0.1 mM rhamnose and, after induction, with 2% (v/v) ethanol. Due to lack of Mn^2+^ in lysogeny broth (Anjem et al., 2009), MnCl_2_ at 0.5 mM was added to cultures expressing 40015 as indicated in corresponding figure captions. Due to low yield of soluble heterologous protein, expression of BOSEA1005_40015, 40016, and 40030 genes was not routinely monitored by gel electrophoresis but by employing shotgun proteomics. Crude extracts from respective transformed *E. coli* strains obtained after heterologous expression were analyzed according to Schopper et al. (2017) with some modifications. Protein extracts were denatured by 5% (w/v final concentration) sodium deoxycholate (≥98%, Sigma-Aldrich) and sequentially treated with 12 mM dithiothreitol and 40 mM 2-iodacetamide before overnight digestion with trypsin (Promega). The desalting method for the digested peptides and also the nano-LC-MS/MS analysis were from Ding and Adrian (2020). Relative abundance of the heterologous BOSEA1005_40015, 40016, and 40030 proteins, other pET-28a(+)-TEV-related proteins (LacI and KanR) as well as a selection of endogenous *E. coli* proteins is compared in Supplementary Table S2.

### 2.6. Analytics

ACE, ANSA and acetoacetate were quantified by HPLC (Shimadzu) with a photodiode array detector employing a Nucleosil 100-5 C18 HD column (240/3 mm, Macherey-Nagel) and an eluent at 0.5 mL min^−1^ containing 100 mM NaH_2_PO_4_ and 10 mM tetrabutylammoniumhydrogensulfate, pH 4.5, and an acetonitrile gradient (% v/v): 2 min 7.5, linear increase to 27.5 in 2 min, 6 min 27.5, linear decrease to 7.5 in 2 min, 12 min 7.5. ACE and ANSA peaks were analyzed at 260 nm, acetoacetate at 274 nm.

Identity of enzymatically prepared ANSA was validated by LC–MS/MS (Agilent) operated with electrospray ionization in negative polarity and equipped with a Zorbax Eclipse Plus Rapid Resolution HT-C18 column (100 × 3 mm, 1.8 μm, Agilent) at 30°C. A binary mobile phase at 0.4 mL min^−1^ consisted of 0.2% formic acid and a methanol gradient: 1 min equilibration at 10%, 2 min linear increase to 55%, 7 min linear increase to 95%, 2 min hold at 95%, 0.5 min decrease to 10% and 3.5 min hold at 10%. See Supplementary material for further details.

## 3. Results

### 3.1. Enzymatic ACE hydrolysis in *Bosea* sp. 100-5 proceeds via ANSA to acetoacetate and strongly depends on Mn^2+^ ions

**Figure 2:**
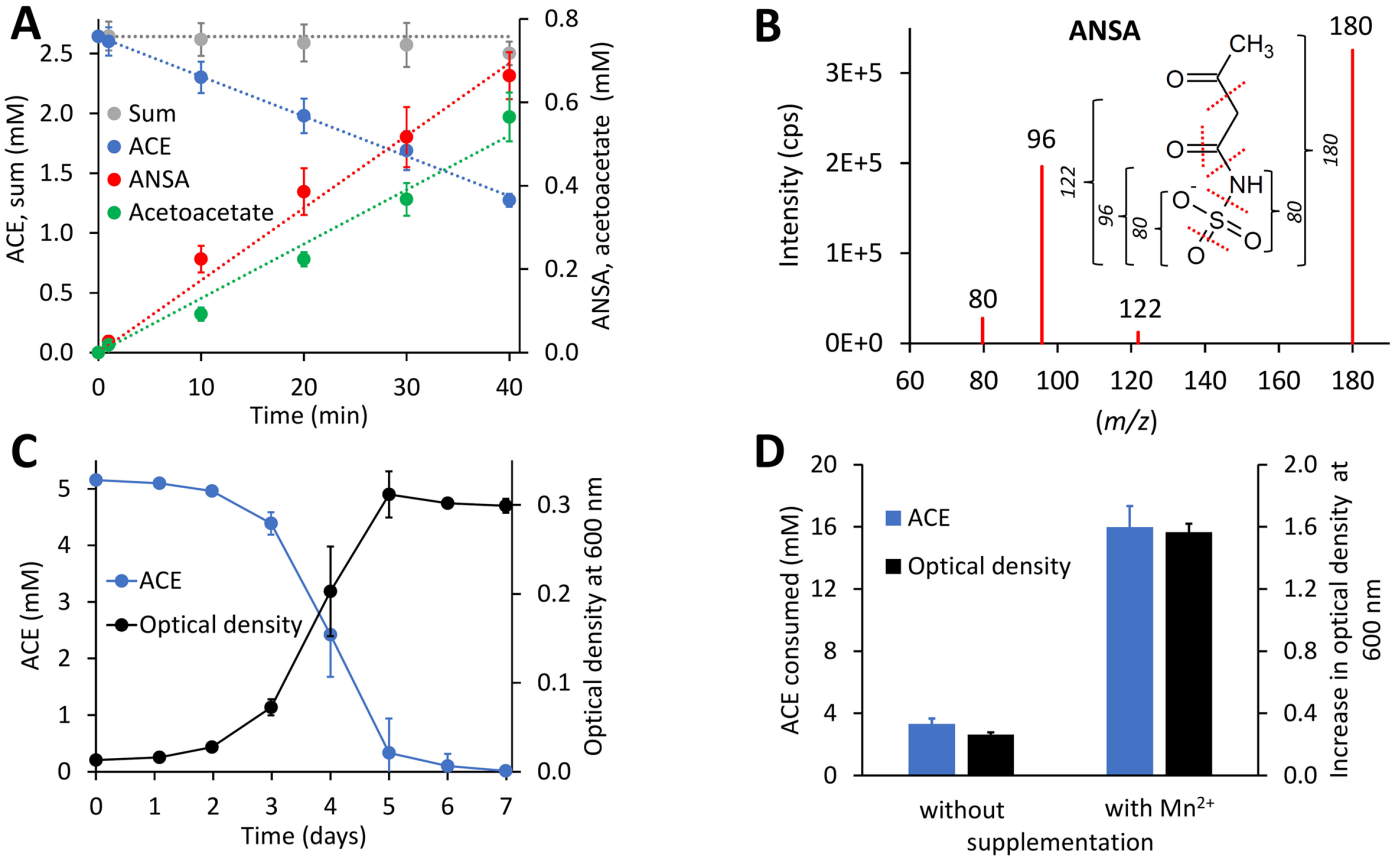
Physiological and biochemical characteristics of bacterial ACE degradation. **(A)** ACE degradation assay at 30°C with crude protein extract (470 μg mL^−1^ total protein) obtained from *Bosea* sp. 100-5 cells grown on ACE as sole source of carbon and energy. **(B)** Identification of ANSA (anion [H_6_C_4_NSO_5_]^−^ with mass-to-charge ratio *m/z* = 180) as product of enzymatic ACE hydrolysis. Enhanced product ion spectrum obtained by LC–MS/MS analysis with electrospray ionization in negative polarity (cps, counts per second). Formation of characteristic products ([SO_3_]^−^ or [H_2_NSO_2_]^−^ with *m/z* = 80, [H_2_NSO_3_]^−^ with *m/z* = 96 and [H_4_C_2_NSO_3_]^−^ with *m/z* = 122) is indicated. **(C)** Batch growth of *Bosea* sp. 100-5 on 5 mM ACE at 30°C in Mn^2+^-rich mineral medium (66.7 μM Mn^2+^) allowing doubling times as low as 19 h. **(D)** Metal dependence of fed-batch growth and degradation of ACE in *Bosea* sp. 100-5 cultivated in Mn^2+^-limited mineral medium (0.15 μM Mn^2+^) without or with Mn^2+^ supplementation (3.7 μM Mn^2+^). Cultures were incubated for 5 days at 30°C with a total amount of 20 mM ACE (see also Supplementary Figure S2A). Values given represent mean and SD of at least five independent experiments.

ACE degradation to acetoacetate via ANSA (Figure 1) was experimentally confirmed with strain *Bosea* sp. 100-5. Protein crude extracts from ACE-grown cells readily degraded ACE to acetoacetate (Figure 2A) with transient accumulation of ANSA (Figure 2B). Enzyme activity did not depend on addition of any cosubstrates, such as NADH or ATP, confirming that ACE degradation to acetoacetate is a two-step hydrolytic reaction as already postulated (Kleinsteuber et al., 2019). Moreover, growth on ACE as sole source of carbon and energy was strongly dependent on supplementation with Mn^2+^ ions. In mineral medium with excess Mn^2+^, ACE degradation allowed exponential growth with doubling times of about 19 h (Figure 2C). In contrast, growth and ACE turnover was substantially reduced in Mn^2+^-limited medium (Figure 2D; Supplementary Figure S2A). Supplementation with other divalent metals, such as Zn^2+^, Fe^2+^, Cu^2+^, or Co^2+^, did not reverse this effect. However, *Bosea* sp. 100-5 grew well in Mn^2+^-limited medium on alternative carbon sources, such as succinate or 3-hydroxybutyrate. The latter substrate is likely metabolized through the same route employed for the carbon skeleton of ACE, i.e., via acetoacetate, acetoacetyl-CoA and acetyl-CoA. Hence, the observed metal dependence is specific for growth on ACE, indicating that one or both hydrolytic steps leading to the intermediate acetoacetate are Mn^2+^-dependent.

### 3.2. Two plasmid-borne gene clusters are involved in ACE degradation

The ACE degradation trait seemed to be unstable in *Bosea* sp. 100-5 and was lost after prolonged cultivation in complex medium without ACE. After several generations in R2A medium, a mutant strain *Bosea* sp. 100-5 Mut1 was isolated that could neither grow on ACE nor consume it in the presence of the alternative carbon source 3-hydroxybutyrate. Moreover, crude extracts did not transform ACE, confirming the lack of the initial hydrolytic enzyme in the mutant.

**Figure 3:**
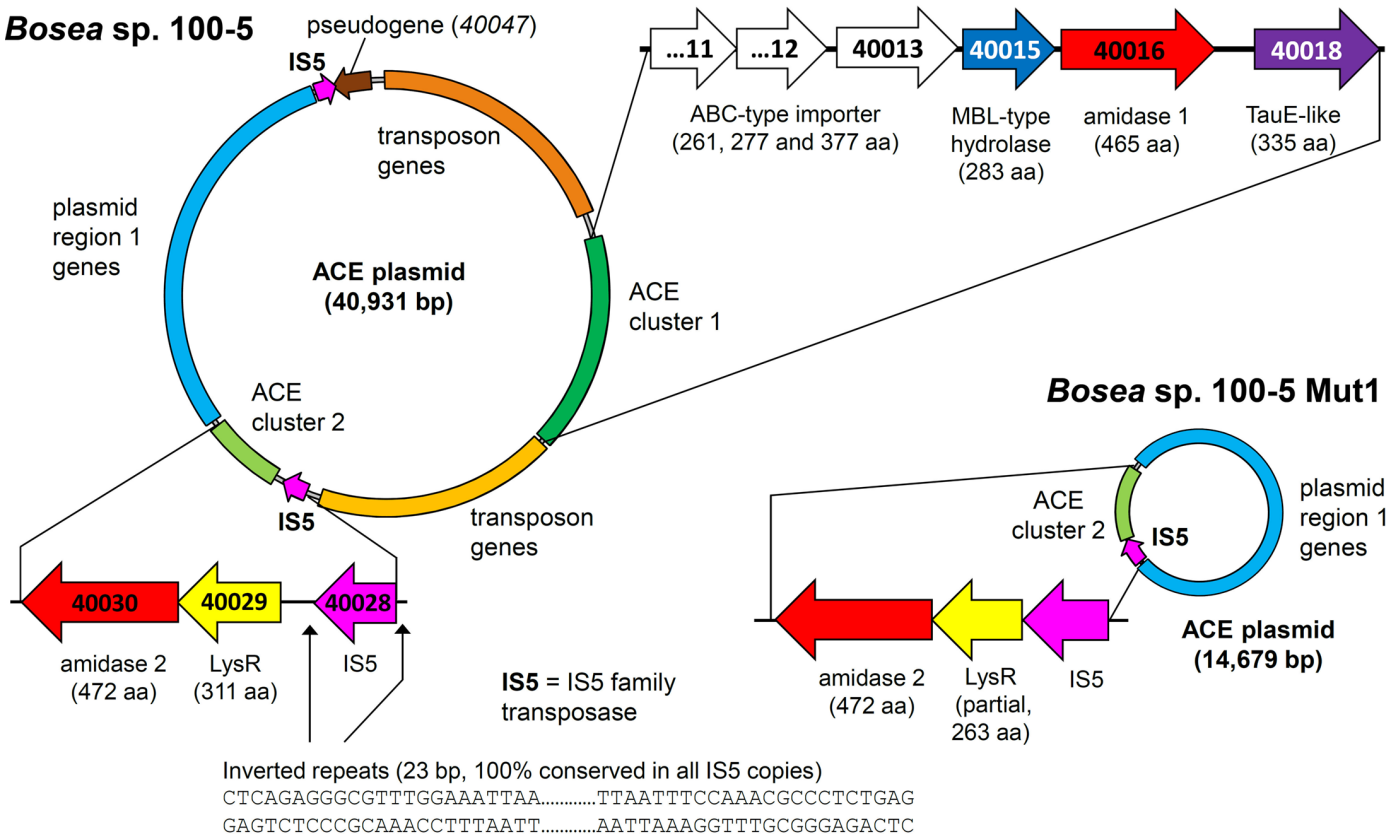
Deletion of the 22.5-kb composite transposon and additional genes from the 40.9-kb ACE plasmid found in *Bosea* sp. 100-5. Within the transposon, the wildtype plasmid bears a gene cluster (ACE cluster 1) encoding two hydrolases and four transport proteins. Additionally, a second gene cluster encoding another hydrolase (ACE cluster 2), other plasmid genes (named “plasmid region 1 genes”) and two copies of an IS5 family transposase (IS5) with inverted repeats as indicated are present. In the mutant strain *Bosea* sp. 100-5 Mut1, a 14.7-kb plasmid containing the genes from plasmid region 1 and ACE cluster 2 remained. The plasmid was manually reconstructed from contigs 56, 57, and 70 of the Mut1 genome sequence (GCA_946047605). Numbers in genes refer to locus tags in *Bosea* sp. 100–5 (prefix BOSEA1005_). Both plasmids are shown true to scale. Detailed map and annotations of the wildtype ACE plasmid can be found in Figure 6 and Table 2, respectively.

Mapping the genome of *Bosea* sp. 100-5 Mut1 on the corresponding wildtype genome revealed that only 2.5% of the nucleotides are missing in the mutant and 98.15% of the CDS are conserved among both strains. The wildtype genome comprises a contig of 40,931 bp that was annotated as a plasmid (Figure 3; Table 2). A gap of 26,252 bp was found in the corresponding region of the mutant genome (Figure 3; Supplementary Figure S3). This deletion comprises a putative metabolic gene cluster of six CDS encoding two hydrolases and four transport proteins, flanked by several CDS typically associated with a composite transposon, such as genes encoding transposases, integrases and transposase-assisting ATPases (Figure 3; Table 2).

**Table 2:** CDS of the ACE plasmid from *Bosea* sp. 100-5 with predicted functions (locus tag prefix BOSEA1005_).

Locus tag	Gene length (bp)	Predicted function of gene product	Plasmid region
40001	1,497^a^	Transposase	Transposon
40002	759	AAA family ATPase	Transposon
40004	777	Putative RNA-directed DNA polymerase	Transposon
40006	1,083	Transposase	Transposon
40007	1,032	Transposase	Transposon
40011	786	ABC type import system, ATP-binding subunit	ACE cluster 1
40012	834	ABC type import system, transmembrane subunit	ACE cluster 1
40013	1,134	ABC type import system, substrate-binding subunit	ACE cluster 1
40015	852	MBL-type hydrolase, ACE sulfatase	ACE cluster 1
40016	1,398	Amidase 1, unknown substrate	ACE cluster 1
40018	1,008	TauE-like small molecular weight anion export system	ACE cluster 1
40019	1,110	Transposase	Transposon
40022	1,512	Transposase	Transposon
40023	756	Insertion sequence ATP-binding protein	Transposon
40025	1,404	Integrase/recombinase	Transposon
40028	783	Transposase	IS5 family element
40029	936	LysR-like transcriptional regulator	ACE cluster 2
40030	1,419	Amidase 2, ANSA amidase	ACE cluster 2
40032	747	Family of unknown function (DUF6118)	Plasmid region 1
40033	3,777	Conjugal transfer protein TraA	Plasmid region 1
40034	291	Mobilization protein, MobC-like	Plasmid region 1
40035	1,779	Type IV secretion system protein TraG	Plasmid region 1
40037	318	Helix-turn-helix protein, antitoxin for 40,038	Plasmid region 1
40038	390	Type II toxin-antitoxin system RelE/ParE family toxin	Plasmid region 1
40039	282	Ribbon-helix–helix protein, CopG family	Plasmid region 1
40040	624	Chromosome partitioning protein ParA	Plasmid region 1
40041	882	Replication protein A	Plasmid region 1
40042	321	Protein of unknown function	Plasmid region 1
40043	252	Protein of unknown function	Plasmid region 1
40044	336	Protein of unknown function	Plasmid region 1
40045	432	Protein of unknown function	Plasmid region 1
40046	783	Transposase	IS5 family element
40047	1,179	low molecular weight anion importer, partial CDS/pseudogene^b^	

a Complete CDS after manually circularizing the contig, identical to the homologous CDS of strain 1 g-11 (CHELA1G11_60037).

b Complete CDS present in strain 1 g-11 (CHELA1G11_60039).

Three CDS of the identified cluster (BOSEA1005_40011, 40012, and 40013) encode the essential components of an ATP-binding cassette (ABC) transport system with BOSEA1005_40011 encoding the ATP-binding protein, BOSEA1005_40012 the transmembrane subunit and BOSEA1005_40013 the periplasmic substrate-binding protein. The two hydrolases encoded by BOSEA1005_40015 and 40016 belong to the metallo beta-lactamase (MBL)-type and amidase signature sequence enzyme families, respectively. BOSEA1005_40018 encodes a protein related to the TauE-like anion transport system, which might be responsible for the removal of the ANSA hydrolysis product sulfamate.

**Figure 4:**
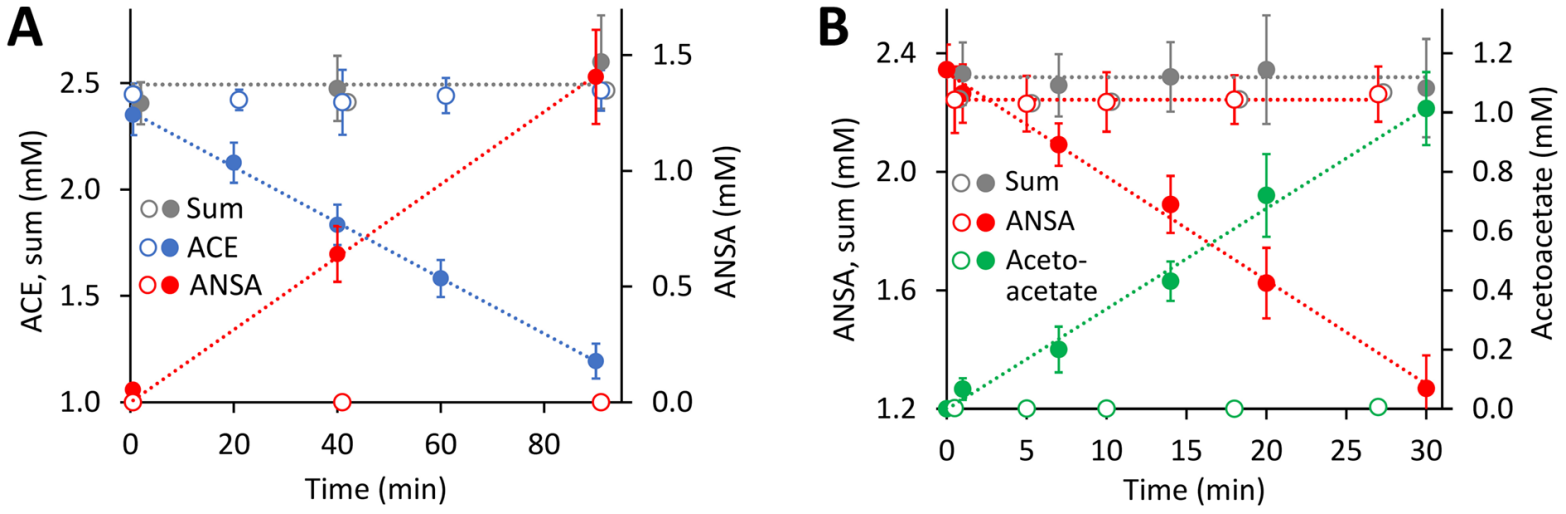
Both hydrolase-encoding gene clusters found on the 40.9-kb ACE plasmid of the wildtype *Bosea* sp. 100-5 are involved in ACE degradation. **(A)** Conversion of ACE to ANSA in crude extracts from *E. coli* Lemo21 (DE3) expressing either BOSEA1005_40016 or 40030 amidase genes (open symbols, 1,330 μg mL^−1^ total protein) or BOSEA1005_40015 hydrolase gene (closed symbols, 1,330 μg mL^−1^ total protein). Expression medium and assay buffer were supplemented with 0.5 and 1 mM MnCl_2_, respectively. ANSA formation from ACE in the presence of the amidases was below 0.2 μM (LC–MS/MS). **(B)** Conversion of ANSA to acetoacetate in crude extracts from *E. coli* Lemo21 (DE3) expressing either BOSEA1005_40016 (open symbols, 74 to 370 μg mL^−1^ total protein) or 40030 amidase (closed symbols, 74 μg mL^−1^ total protein) genes. Acetoacetate formation with BOSEA1005_40016 was below 10 μM (HPLC). Values given represent mean and SD of at least four independent experiments.

Catalytic activity of MBL-type hydrolases depends on divalent metal ions coordinated by highly conserved active site His and Asp residues (Gonzalez, 2021). Considering the observed Mn^2+^ dependence of ACE degradation in *Bosea* sp. 100-5 (Figure 2D; Supplementary Figure S2A), the BOSEA1005_40015 enzyme was the most likely candidate for the initial attack on ACE. This assumption was proven by heterologous expression in *E. coli* (Supplementary Table S2), which also confirmed the Mn^2+^ dependence of the enzyme (Figure 4A; Supplementary Figure S2B). The ACE sulfatase encoded by BOSEA1005_40015 has 283 aa with a predicted molecular weight of 31.9 kDa. Conserved residues for coordination of two divalent metal ions per subunit are H61, H63, D65, H66, H172, and H251.

Enzymes with amidase signature sequence do not show any specific metal dependence but possess a unique Ser Ser Lys (SSK) catalytic triad for the nucleophilic attack of the carbonyl carbon found in amide groups (Shin et al., 2002). BOSEA1005_40016 encodes a protein of 465 aa with a molecular weight of 49.3 kDa bearing the amidase signature sequence in residues K81, S156, and S180. Consequently, it was tempting to assign the hydrolytic activity for ANSA degradation to BOSEA1005_40016. However, directly downstream of the composite transposon, a second metabolic gene cluster was found (Figure 3) encoding a LysR-like transcriptional regulator (BOSEA1005_40029) and a second enzyme of the SSK triad amidase signature family (BOSEA1005_40030, 39% amino acid identity with BOSEA1005_40016). The latter genes are still present in the mutant plasmid (Figure 3). In crude extracts from 3-hydroxybutyrate-grown Mut1 cells, ANSA was readily converted to acetoacetate (Supplementary Figure S4), ruling out the BOSEA1005_40016 enzyme as the primary ANSA amidase. Moreover, although both amidases showed similar relative abundance after heterologous expression (Supplementary Table S2), crude extracts of *E. coli* with heterologous BOSEA1005_40030 protein showed high ANSA-hydrolyzing activity, whereas heterologous BOSEA1005_40016 protein did not confirm ANSA hydrolysis (Figure 4B). Phylogenetic relationships between BOSEA1005_40030 and other amidase genes are illustrated in Supplementary Figure S5. The tree clearly shows that the amidase encoded by BOSEA1005_40016 clusters distantly from the putative ANSA amidase cluster. However, its closest relatives are not characterized and their substrates are unknown.

Taken together, the genome comparison between *Bosea* sp. 100-5 and its deletion mutant Mut1 as well as the heterologous expression of the three hydrolases confirmed that the two-step hydrolysis of ACE to acetoacetate and sulfamate is encoded by two different gene clusters located on one plasmid in *Bosea* sp. 100-5 (Figure 3; Table 2).

### 3.3. The plasmid-borne metabolic genes found in *Bosea* sp. 100-5 are highly conserved among ACE degraders

**Figure 5:**
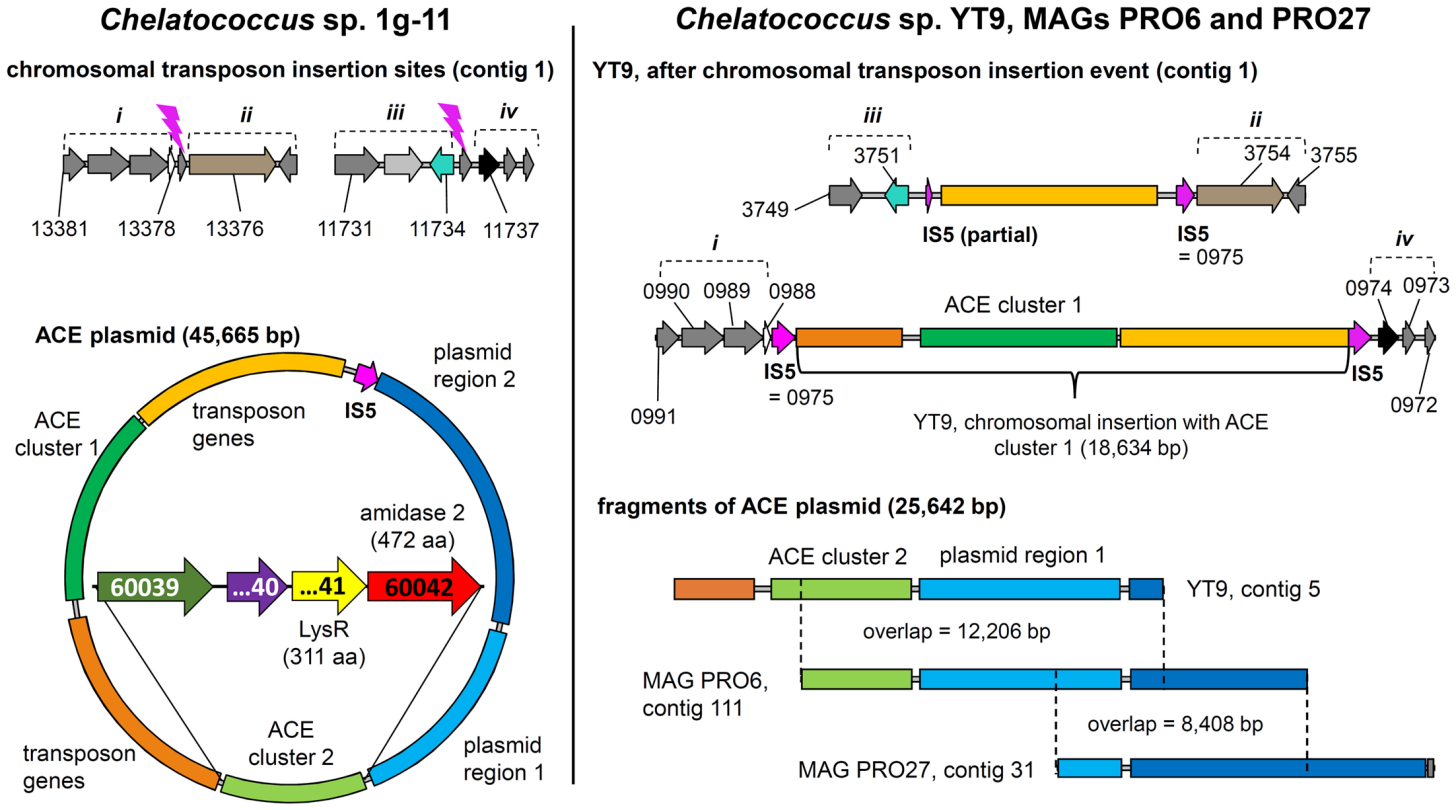
Tracing back the transposition event resulting in the insertion of the transposon bearing the ACE cluster 1 into the chromosome of strain *Chelatococcus* sp. YT9. (Left) *Chelatococcus* sp. 1 g-11, chromosomal insertion sites (assembly accession GCA_930633515, contig 1, locus tag prefix CHELA1G11_) for the IS5 family transposase (IS5) encoded on the 45.7-kb ACE plasmid (contig 6) that also bears the ACE sulfatase and ANSA amidase (“amidase 2”) genes on ACE clusters 1 and 2, respectively. Compared to the corresponding plasmid found in wildtype *Bosea* sp. 100-5 (Figure 3), only a part of the plasmid region 1 genes is shared (“plasmid region 1”), whereas other genes are only present in strain 1 g-11 (“plasmid region 2”). Regions describing the chromosomal rearrangement are labeled *i* to *iv*. Detailed ACE plasmid map and annotations can be found in Supplementary Figure S6. (Right) *Chelatococcus* sp. YT9, chromosomal sites affected by the transposon insertion event (NZ_JAHBRW010000001.1, locus tag prefix WP_21332). At one site, the transposon with ACE cluster 1 is integrated. Due to the insertion event, identical copies of the IS5 family transposase gene frame the whole gene cluster (WP_213320975.1; 100% identical with BOSEA1005_40028 and BOSEA1005_40046 IS5 genes). From the ACE plasmid itself, however, only a small fragment is present in the genome of strain YT9 (NZ_JAHBRW010000005.1). Additional fragments can be found in MAGs (JAHBYS010000111.1, JAHBYU010000031.1) obtained from the ACE-degrading enrichment cultures from which also strain YT9 was isolated. Numbers marking genes refer to locus tags (with prefix as indicated). Plasmids and DNA fragments are shown true to scale. Genes with the same color refer to identical or closely related sequence (≥80% amino acid identity in predicted gene product, in most cases > 99%).

In addition to the previously described strains *Bosea* sp. 100-5, *Bosea* sp. 3-1B, *Chelatococcus* sp. 1 g-11, and *Chelatococcus* sp. 1 g-2 (Kleinsteuber et al., 2019), we isolated several other ACE-degrading *Chelatococcus* strains and one *Shinella* strain from samples collected in WWTPs from different locations in Germany (Table 1) and sequenced the genomes of all these strains. Supplementary Table S1 summarizes the genomic features of the nine ACE degraders and the degradation-defective mutant *Bosea* sp. 100-5 Mut1. All strains have large genomes (5.9 Mb for the *Bosea* strains, 7 to 7.3 Mb for the *Chelatococcus* strains, and 7.8 Mb for *Shinella* sp. WSC3-e). Five other ACE-degrading strains isolated from a WWTP in Hong Kong (Huang et al., 2021, 2022) were included in genome comparison (Table 1). In agreement with their proposed role in ACE degradation, the two gene clusters were found in all genomes on plasmid-like contigs ranging from 41,507 to 45,942 bp, except for the *Shinella* strains and strain HY11, which harbor only ACE cluster 2, and strain YT9, which has both clusters but only cluster 2 on a plasmid (Table 1). While ACE cluster 1 (sulfatase cluster) together with its flanking transposon-related CDS is highly conserved (>99% nucleotide identity) among all these strains, ACE cluster 2 (amidase cluster) is variable in size (two to four CDS) but always contains the conserved CDS for the ANSA amidase and the LysR-like transcriptional regulator. The ACE plasmid of *Chelatococcus* sp. 1 g-11 is exemplarily shown in Figure 5 (see Supplementary Figure S6 for a detailed plasmid map and annotations).

### 3.4. Putative ACE degradation gene clusters in public sequence databases

**Figure 6:**
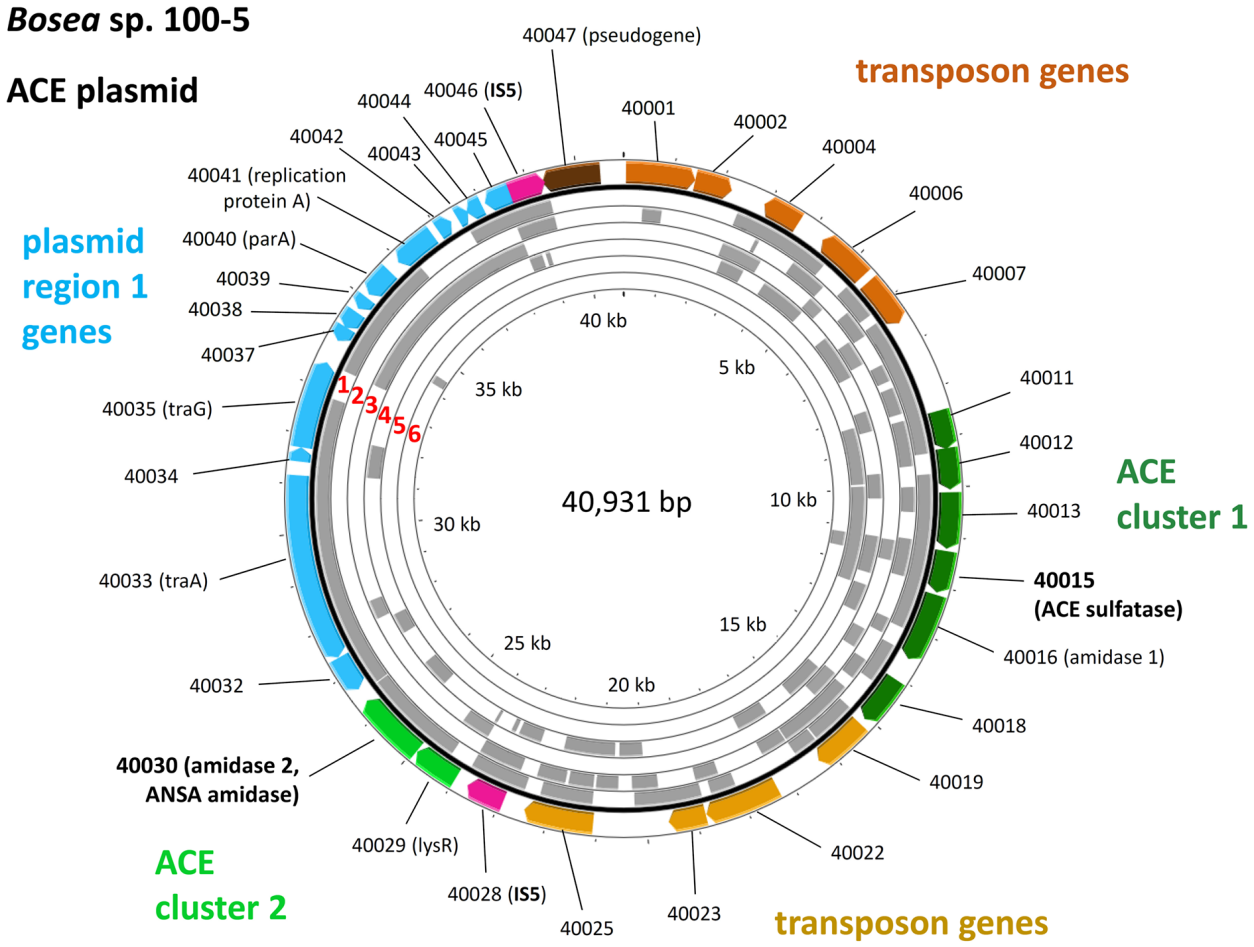
Mapping of the ACE plasmid genes on metagenome and metatranscriptome datasets associated with wastewater, bioreactors and WWTPs. These findings indicate the worldwide distribution and genetic conservation of the ACE degradation gene clusters and the ACE plasmid. The outer colored ring shows the genes on the *Bosea* sp. 100-5 ACE plasmid (color code as in Figure 3; locus tag prefix is BOSEA1005_). For complete annotation, see Table 2. Gray areas show hits (≥98% nucleotide identity) within the following metagenome and metatranscriptome datasets (numbering highlighted in red): 1 = Anaerobic bioreactor from Hunan University (sample 11), China; 2 = Wenshan WWTP, Taiwan; 3 = Linkou WWTP, Taiwan; 4 = WWTP in Virginia, United States; 5 = Weurt WWTP, The Netherlands; 6 = Klosterneuburg WWTP (sample MT KNB_C2_LD), Austria. For complete matches from datasets, see Supplementary Table S3. The figure was generated with Proksee.

The ACE sulfatase (BOSEA1005_40015) appears to be a unique feature of the pathway. While its gene is ≥99% conserved among all genome-sequenced ACE degraders thus far (Table 1), only very distantly related sequences (with <30% amino acid identity at ≥80% coverage) were found in other published genomes (bacterial isolates and MAGs). Therefore, we surveyed public metagenomes and metatranscriptomes from wastewater environments and found the ACE sulfatase gene in sequence datasets from activated sludge sampled in Austria, China, Germany, Netherlands, Taiwan, and USA (Figure 6). As the samples were collected between 2015 and 2018, the first record of the ACE sulfatase gene in 2015 can be traced back to the Klosterneuburg WWTP in Austria (Supplementary Table S3).

In some datasets that presented the ACE sulfatase gene, other elements of the ACE plasmid from *Bosea* sp. 100-5 were also found. In the metatranscriptome from the Weurt WWTP in Nijmegen (Netherlands), genes of ACE cluster 1 corresponding to BOSEA_40011 to 40016 were detected, and the metagenome from the Wenshan WWTP (Taiwan) was shown to contain fragments of all ACE cluster 1 genes. In the latter dataset, we also found contigs related to the transposon and other genes of the *Bosea* sp. 100-5 ACE plasmid (Figure 6). While likewise including fragments of 40012 and 40016 of cluster 1, the metagenome from the Virginia WWTP is one of the two datasets representing part of the 40030 gene of ACE cluster 2, and a particularly high coverage of 75% of the *Bosea* sp. 100-5 ACE plasmid was detected in the dataset from activated sludge microbial communities from an anaerobic bioreactor operated at Hunan University (Changsha, China).

## 4. Discussion

The artificial sweetener ACE was once considered recalcitrant in WWTPs (Buerge et al., 2009) but recently its emerging biodegradability was reported (Cardenas et al., 2016; Castronovo et al., 2017; Kahl et al., 2018). The earliest indication for biodegradation based on ACE monitoring in wastewater was found in WWTPs located in Queensland, Australia (Cardenas et al., 2016) and Eriskirch, Germany (Castronovo et al., 2017), demonstrating removal efficiencies of about 90% in the sampling campaign years 2012 and 2013, respectively. Microbial community surveys highlighted the importance of *Alphaproteobacteria* for ACE degradation (Kahl et al., 2018; Huang et al., 2021). In particular, representatives of the genera *Bosea*, *Chelatococcus* and *Shinella* capable of degrading ACE were isolated recently (Kleinsteuber et al., 2019; Huang et al., 2021, 2022). Our present study revealed the enzymatic and genetic background of ACE degradation and offers a glimpse of the evolutionary mechanisms driving the fast and worldwide spread of this novel xenobiotics degradation trait.

The comparison of the wildtype *Bosea* sp. 100-5 and its ACE degradation-defective mutant clearly revealed the involvement of a plasmid-borne gene cluster (ACE cluster 1) encoding an MBL-type hydrolase (BOSEA1005_40015) in ACE degradation. The heterologous expression of the latter enzyme corroborates its function as ACE sulfatase. Recently, Castronovo et al. (2023) confirmed the presence of the MBL-type hydrolase in the metaproteome of an ACE-degrading enrichment culture dominated by *Chelatococcus*. Accordingly, the complete gene cluster including the flanking transposon-related CDS is highly conserved among the ACE-degrading strains analyzed in the present study. However, BOSEA1005_40015 appears to be unique and unprecedented, as the gene was not found in any other genomic context, and no closely related enzyme has been characterized thus far. In analogy to the distantly related hydrolases attacking phosphate ester bonds in nucleotides, the BOSEA1005_40015 enzyme might attack the sulfonyloxy group in ACE. Substantial sequence coverage with the BOSEA1005_40015 protein was only found for Apyc1 from *Bacillus* phage BSP38 (PDB ID 7 T28) showing 26% identity (at 90% query coverage). The phage protein catalyzes the hydrolysis of cyclic mononucleotides involved in the antiphage defense system of its host. Its di-metal center is occupied by Zn^2+^ ions (Hobbs et al., 2022). Other distantly related hydrolases (only <30% identical residues at >80% query coverage) are often annotated as the short form of ribonuclease Z (consisting of 300 to 400 aa) that removes extra 3′ nucleotides from tRNA precursors (Takaku et al., 2004). From the specific metal dependence of the ACE degradation observed in *Bosea* sp. 100-5 (Figure 2D; Supplementary Figure S2A) and the heterologous BOSEA1005_40015 enzyme (Figure 4A; Supplementary Figure S2B), it can be concluded that the reaction center of the active MBL-type hydrolase is exclusively occupied by two Mn^2+^ ions. This is somewhat surprising, as MBL-type enzymes may be able to coordinate other divalent metal ions when the favored species is not available (Hu et al., 2009). Presumably, in the case of the ACE sulfatase, the substitution with other metals than Mn^2+^ results in a substantial reduction in activity. This might be explained by the ACE structure that obviously requires the full activity of the enzyme, as a nucleophilic attack toward the sulfur or C3 atom (Figure 1) is quite challenging due to the electron-richness of the ring system.

**Figure 7:**
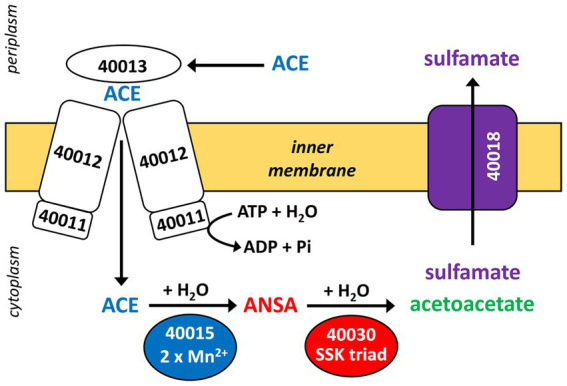
Proposed scheme of the two-step hydrolysis of ACE catalyzed by cytoplasmic MBL-type hydrolase BOSEA1005_40015 (from ACE cluster 1) and amidase BOSEA1005_40030 (from ACE cluster 2). Likely, ACE and its final hydrolysis product sulfamate are taken up and removed, respectively, by the transport systems encoded in ACE cluster 1 (see Figure 3), but both assumptions need experimental support. Numbers shown refer to locus tags in *Bosea* sp. 100–5 (prefix BOSEA1005_).

The required translocation of the negatively charged substrate into the cell across the cytoplasmic membrane and against the membrane potential is likely enabled by the ABC-type transporter encoded in ACE cluster 1 (Figures 3, 7). Protein sequences of the importer are distantly related to uptake systems associated with the transport of small inorganic or organic anions. However, only very low identity to thus far studied proteins can be found, e.g., the periplasmic substrate-binding protein shows 27% sequence identity at 18% query coverage to TauA from *E. coli* (UniProt ID Q47537), which was reported to bind preferentially the short-chain alkanesulfonate taurine (Qu et al., 2019). Finally, as the ACE hydrolysis product sulfamate formed in the cytoplasm is not used by ACE-degrading bacteria (Kleinsteuber et al., 2019), an export system is probably needed for the efficient removal of the waste product, which could be the BOSEA1005_40018 gene product. This protein belongs to the TauE/SafE family of transmembrane proteins involved in the export of small anions, such as sulfite, sulfoacetate, and 3-sulfolactate (Weinitschke et al., 2007; Mayer et al., 2012). Beyond this finding, the TauE/SafE system is not well characterized and the amino acid sequence is poorly conserved among representatives of the protein family, e.g., BOSEA1005_40018 does not show significant similarity (blastp) with WP_011617520.1 from *Cupriavidus necator* H16.

Together with BOSEA1005_40016 encoding an amidase, the gene cluster seems to be dedicated for the two-step hydrolytic degradation of a substrate sharing some features with ACE. However, BOSEA1005_40016 was lost in *Bosea* sp. 100-5 Mut1 but protein extracts still hydrolyzed ANSA. Moreover, heterologous expression of another amidase gene (BOSEA1005_40030) in *E. coli* identified its function in ANSA hydrolysis and disproved the involvement of BOSEA1005_40016. Related sequences of BOSEA1005_40016 (about 60% identity at 96% query coverage) are present in gene environments other than the ACE degradation gene cluster 1, e.g., WP_013973238.1 (nicotine-degrading *Pseudomonas putida* S16) and WP_244145082.1 (root nodule bacterium *Paraburkholderia tuberum* LMG 21444). However, as these matches lack biochemical characterization, no conclusion on substrate preferences can be drawn. Therefore, the genetic makeup of ACE cluster 1 implies that it originally evolved for a different function than ACE degradation.

The ANSA amidase gene BOSEA1005_40030 is located in a second gene cluster on the ACE plasmid. In *Bosea* sp. 100-5, this cluster only consists of CDS for a transcriptional regulator (LysR) and the ANSA amidase. In *Chelatococcus* sp. 1 g-11, additional genes encoding an anion import system (CHELA1G11_60039) and another TauE/SafE-like exporter (CHELA1G11_60040) are present (Figure 5; Supplementary Figure S7). In contrast to BOSEA1005_40015, sequences closely related to BOSEA1005_40030 can be found in genomes of bacteria that do not degrade ACE, e.g., WP_176954172.1 from *Paraburkholderia sartisoli* LMG 24000 showing 66% amino acid identity at 99% coverage. Interestingly, the gene clusters in strains 1 g-11 and LMG 24000 do not only share the amidase gene but also that encoding the anion import system (75% amino acid identity at 93% coverage; Supplementary Figure S7). Additionally, a TauE/SafE system is present in strain LMG 24000, albeit not closely related to the ones encoded on the ACE plasmids. This constellation appears to have evolved for uptake of an extracellular amide that is hydrolyzed in the cytoplasm. *Bosea* sp. 100-5 lacks the TauE/SafE system, likely due to insertions of the IS5 family transposase and subsequent rearrangements, and the anion transport gene is only present as a pseudogene outside ACE cluster 2 (Figure 3; Table 2). Hence, only the BOSEA1005_40030 amidase is needed for ANSA degradation but not the anion import system present in strain *Chelatococcus* sp. 1 g-11.

Intriguingly, ACE cluster 1 has been found only in the genera *Bosea* and *Chelatococcus* thus far. The specific physiological background making these genera suitable hosts of ACE cluster 1 could be related to their ability to accumulate high intracellular manganese concentrations (Fredrickson et al., 2008). On the other hand, the genomes of recently isolated *Shinella* strains harbor only ACE cluster 2 (Table 1), which might imply the involvement of other sulfatases catalyzing the initial hydrolysis reaction in these strains.

Compounds structurally related to ACE that could be likewise attacked by the MBL-type hydrolase are other natural or synthetic sulfamic acid derivatives (Spillane and Malaubier, 2014; Awakawa et al., 2021), in particular, O- and N-substituted sulfamates. In this context, Supplementary Figure S8A proposes a two-step enzymatic hydrolysis of ascamycin and other natural or synthetic aminoacyl sulfamate ribonulceoside antibiotics (Isono et al., 1984), involving sulfatase and amidase activities. Also sulfonamide antibiotics might be relevant, albeit having a deviating structure compared to sulfamates, as the sulfur atom of the sulfonyl group is directly linked to an aniline ring system (Supplementary Figure S8B). Although bacterial degradation seems to proceed mainly via ipso-hydroxylation leading to the decomposition of the resulting intermediates and the release of sulfite (Ricken et al., 2013), alternatively, enzymatic hydrolysis by attacking the sulfur atom of, e.g., sulfacetamide, might lead to the release of aniline and an ANSA-related N-sulfonated acyl amide (Supplementary Figure S8B). In support, aniline as degradation product not compatible with the ipso-hydroxylation mechanism has been reported for *Pseudomonas psychrophila* HA-4 incubated with the sulfonamide antibiotic sulfamethoxazole (Jiang et al., 2014). Interestingly, the latter and other sulfonamides are partially removed from municipal wastewater concomitantly with ACE (Yang et al., 2017).

**Figure 8:**
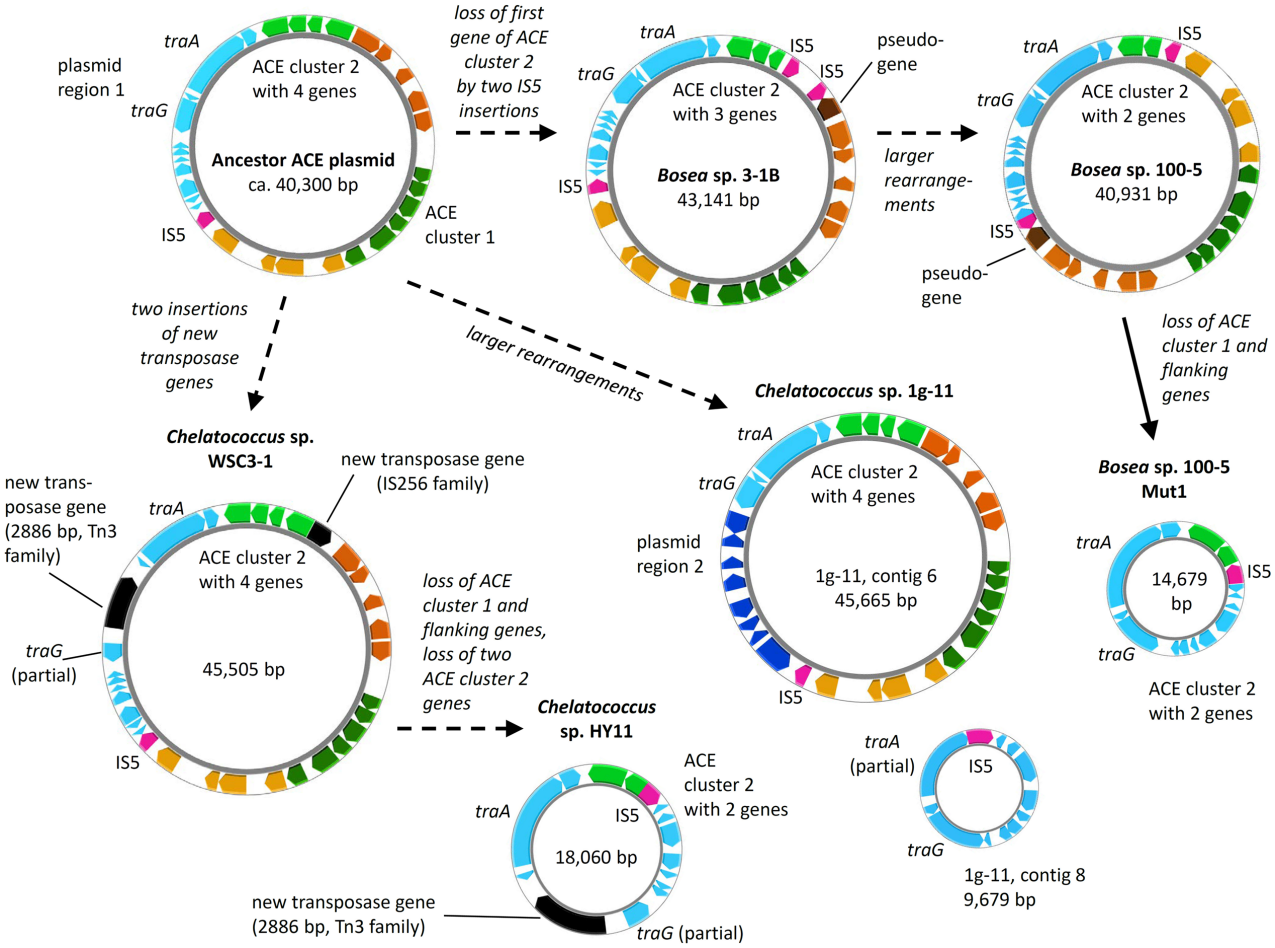
Diversity of ACE plasmids in isolated *Bosea* and *Chelatococcus* strains. Inspection of the ACE cluster 2 genes and a plasmid region consisting of genes encoding conjugative transfer (e.g., *traA* and *traG*) and other functions (“plasmid region 1”) indicates a possible ancestor closely related to the plasmids from *Bosea* sp. 3–1B and *Chelatococcus* sp. WSC3-1. Only in *Chelatococcus* sp. 1 g-11, plasmid region 1 is substantially smaller, while new genes (“plasmid region 2”) not present in the other plasmids are found. However, strain 1 g-11 still harbors an almost complete region 1 on a separate plasmid (contig 8), deviating only by a new insertion of the IS5 family element (IS5) in the Ti-type conjugative transfer relaxase gene *traA*. On the other hand, ACE plasmids of strains WSC3-1 and HY11 share the disruption of the type IV secretory system conjugative DNA transfer family protein gene *traG* by a 2,886-bp Tn3 family transposase gene (WP_113946498) not present in the other plasmids. Dashed arrows propose the plasmid evolution mainly induced by the IS5 element, whereas the event indicated by the solid arrow was demonstrated experimentally in this study. Gene rearrangements might include transpositions into the chromosome as shown in Figure 5. Smaller plasmids (<20 kb) are not true to scale but about 150% to 200% enlarged. Color code as used in Figure 5. The figure was generated with Proksee.

Metabolic genes embedded in transposons and their location on a conjugative plasmid are typical features of bacterial pathways involved in degradation of xenobiotics, such as antibiotics and pesticides (Springael and Top, 2004). This enables the evolution and distribution of novel degradation pathways or other functional traits that facilitate niche adaptation. In case of ACE, mediated by the IS5 family transposase, ACE cluster 1 can easily transpose between different replicons (Figure 5), which might have supported the recruitment of metabolic genes for ACE degradation. A potential scenario of the genetic rearrangements that might have led from an ancestral plasmid to the ACE plasmids currently found in the various *Bosea* and *Chelatococcus* strains is illustrated in Figure 8. In the mutant strain *Bosea* sp. 100-5 Mut1, the composite transposon comprising ACE cluster 1 has been completely deleted from the plasmid. A similar situation was found in the published genome of the ACE-degrading strain *Chelatococcus* sp. HY11 isolated from a WWTP in Hong Kong (Huang et al., 2021). Here, ACE cluster 1 is also missing, like most of the other CDS of the composite transposon. However, some other genes corresponding to the ACE plasmid are located on a short contig of the HY11 genome (JAHBRX010000006.1). Likewise, various fragments of the rudimentary plasmid, lacking most of the 26-kb region associated with ACE hydrolysis, are present in the MAG sequences PRO6 and PRO27 (Figure 5). The corresponding metagenome was obtained from samples of ACE-degrading consortia seeded with activated sludge from the WWTP in Hong Kong from which also the ACE degraders *Chelatococcus* sp. HY11 and YT9 were isolated (Huang et al., 2021). It is worth mentioning that the gene for the type IV secretion system protein TraG (Figure 8; Table 2) is disrupted by a Tn3 family transposase gene (WP_113946498) in strain HY11, indicating some recombination events in this genomic region that identically occurred in our strain *Chelatococcus* sp. WSC3-1 isolated from a WWTP located in Leipzig, Germany (Table 1).

While the genome sequences of *Bosea* sp. 100-5 Mut1 and likely also of *Chelatococcus* sp. HY11 document the loss of ACE cluster 1, the genome of *Chelatococcus* sp. YT9 gives evidence for the insertion of the composite transposon into the chromosome. Here, ACE cluster 1 is flanked by almost all transposon components found in the 26 kb region of *Bosea* sp. 100-5 (Figure 5). As the insertion sites are well conserved in *Chelatococcus* sp. 1 g-11, the event can be reconstructed. Likely, the mobile element was originally located on a plasmid as the ones found in our ACE-degrading isolates (Figure 8). Accordingly, short contigs of strain YT9 and MAGs PRO6 and PRO27 harbor parts of the transposon genes, ACE cluster 2 and some other genes of the ACE plasmid found in *Chelatococcus* sp. 1 g-11 (Figure 5). The genome rearrangements in *Bosea* and *Chelatococcus* strains illustrate the genetic plasticity of the ACE degradation trait and suggest that the pathway recruitment is not yet completed since the ANSA amidase gene is located in another plasmid-borne cluster.

From the biochemist’s perspective, ACE represents a challenging molecule combining features of a carboxylic acid amide and a sulfamic acid ester in an electron-rich ring system. Particularly, nucleophilic attack of the amide is difficult due to the localization of the negative charge mainly at the nitrogen atom (Popova et al., 2012). Furthermore, even when used for productive degradation, growth yields on ACE would be low, as (i) its uptake likely depends on ATP hydrolysis and (ii) only the carbon skeleton could be used for assimilation and dissimilation (representing only 50% of the molecular weight). Hence, it can be concluded that nature lacked an appropriate degradation mechanism when ACE was introduced into the environment for the first time in the 1980s (Klug and von Rymon Lipinski, 2012). In agreement, the artificial sweetener was long time considered recalcitrant against enzymatic attack (Buerge et al., 2009). The unprecedented high removal efficiency monitored in WWTPs since 2012 (Cardenas et al., 2016) points to the evolution of an ACE degradation pathway within a 30-years period. However, considering its low concentration in wastewater [typically <100 μg L^−1^ in Europe, China and Australia (Buerge et al., 2009; Gan et al., 2013; Cardenas et al., 2016; Castronovo et al., 2017)], ACE is a rather poor substrate. Consequently, its utilization is likely not a sufficient driving force for the evolution of such a well-coordinated degradation pathway as depicted in Figure 7. Rather, co-metabolism with more dominant environmental chemicals can be expected (Kennes-Veiga et al., 2022). In line with this, no earlier stage or any other evolution of ACE cluster 1 was traceable in genomes and metagenomes. However, older metagenome datasets might have insufficient sequencing depth to cover such rare genes. Moreover, MAGs are prone to miss variable genes of natural populations (Meziti et al., 2021). Accordingly, matches for the genes of the ACE clusters were only found in partially assembled metagenome and metatranscriptome data (Figure 6; Supplementary Table S3) but not in MAGs. Direct and systematic Sequence Read Archive (SRA) search would be needed for tracing gene fragments that are discarded by assembly algorithms. As exemplarily shown for the metagenome dataset from the WWTP in Virginia (Figure 6; Supplementary Table S3), we could map SRA datasets on the entire ACE clusters (Supplementary Figures S9, S10). Mapping of another SRA dataset from a WWTP in Christchurch, New Zealand, on ACE cluster 1 and 2 sequences is illustrated in Supplementary Figure S11.

The evolution and distribution of the individual ACE clusters likely started much earlier than in the 2010s, possibly triggered by other natural or anthropogenic chemicals that exerted higher selective pressure than the non-toxic ACE. However, the last evolutionary step of combining the two gene clusters in one genome can be attributed to the recent bacterial adaptation to ACE as energy and carbon source. Due to the low ACE concentration in wastewater, this evolution might have occurred in rather oligotrophic environments, such as the late sections of treatment wetlands (Kahl et al., 2017, 2018) and biofilms thriving in receiving waters. The concomitant occurrence of identical gene clusters in Germany and China (Figures 5, 6) suggests that the ACE plasmid formed first at one site and then was distributed rapidly among WWTPs. Further isolation studies and molecular surveys using the ACE and ANSA hydrolases as genetic markers could reveal the origin of the ACE degradation trait and how it has spread in aquatic environments worldwide.

## Data availability statement

The datasets presented in this study can be found in online repositories. The names of the repository/repositories and accession number(s) can be found in the article/Supplementary material.

## Author contributions

SK, TRo, TRe, LA, and CD conceived and directed the project. DP, MB, K-PL, CS, TRo, YL, and CA performed the experiments and were involved in method design as well as data analysis. MB, TRo, and SK wrote the manuscript with substantial input of MB, TRo, DP, YL, CA, CS, K-PL, CD, TRe, LA, and SK. All authors contributed to the article and approved the submitted version.

## Funding

Cloud computing facilities used for the bioinformatics analyses were provided by the BMBF-funded de.NBI Cloud within the German Network for Bioinformatics Infrastructure (de.NBI) (031A537B, 031A533A, 031A538A, 031A533B, 031A535A, 031A537C, 031A534A, and 031A532B). YL wishes to thank the CSC for the doctoral fellowship (202004910433).

## Conflict of interest

The authors declare that the research was conducted in the absence of any commercial or financial relationships that could be construed as a potential conflict of interest.

## Publisher’s note

All claims expressed in this article are solely those of the authors and do not necessarily represent those of their affiliated organizations, or those of the publisher, the editors and the reviewers. Any product that may be evaluated in this article, or claim that may be made by its manufacturer, is not guaranteed or endorsed by the publisher.

## Footnotes

^1^https://github.com/rrwick/Filtlong

^2^https://img.jgi.doe.gov
