# Supplementary material for: Recently evolved combination of unique sulfatase and amidase genes enables bacterial degradation of the wastewater micropollutant acesulfame worldwide
Source: Front Microbiol. 2023 Jul 27;14:1223838. doi: 10.3389/fmicb.2023.1223838 (PMC10413263; doi:10.3389/fmicb.2023.1223838)
Supplement: Supplementary file 1 [file Data_Sheet_1.PDF]

Supplementary Material

Recently evolved combination of unique sulfatase and amidase genes enables bacterial degradation of the wastewater micropollutant acesulfame worldwide

Maria L. Bonatelli, Thore Rohwerder*, Denny Popp, Yu Liu, Caglar Akay, Carolyn Schultz, Kuan-Po Liao, Chang Ding, Thorsten Reemtsma, Lorenz Adrian, Sabine Kleinsteuber*

*** Correspondence:** Address correspondence to Thore Rohwerder (thore.rohwerder@ufz.de), Sabine Kleinsteuber (sabine.kleinsteuber@ufz.de)

This Supplementary Data contains:

1 Description of mineral salt medium DSMZ 461

2 Description of mineral salt medium DSMZ 462

3 Details on bacterial genome sequencing results (Table S1)

4 Relative abundance of heterologous proteins in *E. coli* crude extracts (Table S2)

5 NCBI and JGI database search for the BOSEA1005_40015 gene (Table S3)

6 Detailed description of the LC-MS/MS method for monitoring ANSA

7 Preparation of ANSA using chromatographically enriched ACE sulfatase

8 Calibration factor for HPLC-based ANSA quantification (Fig. S1)

9 Mn^{2+} dependence of *Bosea* sp. 100-5 and MBL-type hydrolase for ACE degradation (Fig. S2)

10 Characterization of mutant strain *Bosea* sp. 100-5 Mut1 (Figs. S3 and S4)

11 Functional assignment for predicted proteins encoded in ACE cluster 2 (Fig. S5)

12 Map and annotations of the ACE plasmid found in strain *Chelatococcus* sp. 1g-11 (Fig. S6)

13 Comparison of gene clusters bearing the gene for the ANSA amidase (Fig. S7)

14 Possible two-step enzymatic hydrolysis of ACE-related chemicals (Fig. S8)

15 Blastn results with ACE cluster 1 and 2 sequences as query against metagenome assembly GCA_009612895.1 and corresponding SRA datasets (Figs. S9 and S10)

16 Blastn results with ACE cluster 1 and 2 sequences as query against SRA datasets of BioProject PRJNA904380 (Figs. S11)

17 References

1 Description of mineral salt medium DSMZ 461

Culture medium recipe available at https://bacmedia.dsmz.de/medium/461.

Complete medium

Compound	Amount	Unit	Conc. [g/L]	Conc. [mM]
Distilled water	1000.00	mL	-	-
Na_2HPO_4 x 2 H_2O	1.45	g	1.441	10.153
KH_2PO_4	0.25	g	0.249	1.826
$CaCl_2$	0.01	g	0.01	0.09
$MgSO_4$ x 7 H_2O	0.50	g	0.497	2.017
$MnSO_4$	0.01	g	0.01	0.066
NH_4Cl	0.30	g	0.298	5.575
NaCl	0.05	g	0.05	0.85
Vitamin solution	5.00	mL	-	-
Trace element solution SL-10	1.00	mL	-	-

Vitamin solution

Compound	Amount	Unit	Conc. [g/L]	Conc. [mM]
Vitamin B_{12}	50	mg	0.05	0.037
Pantothenic acid	50	mg	0.05	0.228
Riboflavin	50	mg	0.05	0.133
Pyridoxamine hydrochloride	10	mg	0.01	0.049
Biotin	20	mg	0.02	0.082
Folic acid	20	mg	0.02	0.045
Nicotinic acid	25	mg	0.025	0.203
Nicotine amide	25	mg	0.025	0.205
alpha-Lipoic acid	50	mg	0.05	0.242
p-Aminobenzoic acid	50	mg	0.05	0.365
Thiamine-HCl x 2 H_2O	50	mg	0.05	0.134
Distilled water	1000	mL		

Trace element solution SL-10

Compound	Amount	Unit	Conc. [g/L]	Conc. [mM]
HCl (25%)	10.0	mL	2.5	68.568
$FeCl_2$ x 4 H_2O	1.5	g	1.5	7.545
$ZnCl_2$	70.0	mg	0.07	0.514
$MnCl_2$ x 4 H_2O	100.0	mg	0.1	0.505
H_3BO_3	6.0	mg	0.006	0.097
$CoCl_2$ x 6 H_2O	190.0	mg	0.19	1.463
$CuCl_2$ x 2 H_2O	2.0	mg	0.002	0.012
$NiCl_2$ x 6 H_2O	24.0	mg	0.024	0.185
Na_2MoO_4 x 2 H_2O	36.0	mg	0.036	0.149
Distilled water	990.0	mL	-	-

The final pH of the medium is 7.5.

2 Description of mineral salt medium DSMZ 462

Culture medium recipe available at https://bacmedia.dsmz.de/medium/462.

Complete medium

Compound	Amount	Unit	Conc. [g/L]	Conc. [mM]
Na_2HPO_4	2.44	g	2.44	17.188
KH_2PO_4	1.52	g	1.52	11.17
$(NH_4)_2SO_4$	0.50	g	0.5	3.784
$MgSO_4$ x 7 H_2O	0.20	g	0.2	0.811
$CaCl_2$ x 2 H_2O	0.05	g	0.05	0.34
Trace element solution SL-4	10.00	mL	-	-
Vitamin solution (Schlegel)	2.50	mL	-	-
Distilled water	1000.00	mL	-	-

Vitamin solution (Schlegel)

Compound	Amount	Unit
p-Aminobenzoate	1.0	mg
Biotin	0.2	mg
Nicotinic acid	2.0	mg
Thiamine-HCl x 2 H_2O	1.0	mg
Calcium pantothenate	0.5	mg
Pyridoxamine	5.0	mg
Vitamin B_{12}	2.0	mg
Distilled water	100.0	mL

Trace element solution SL-4

Compound	Amount	Unit
EDTA	0.5	g
$FeSO_4$ x 7 H_2O	0.2	g
Trace element solution SL-6	100.0	mL
Distilled water	900.0	mL

Trace element solution SL-6

Compound	Amount	Unit	Conc. [g/L]	Conc. [mM]
$ZnSO_4$ x 7 H_2O	0.10	g	0.1	0.348
$MnCl_2$ x 4 H_2O	0.03	g	0.03	0.152
H_3BO_3	0.30	g	0.3	4.851
$CoCl_2$ x 6 H_2O	0.20	g	0.2	1.54
$CuCl_2$ x 2 H_2O	0.01	g	0.01	0.059
$NiCl_2$ x 6 H_2O	0.02	g	0.02	0.154
Na_2MoO_4 x 2 H_2O	0.03	g	0.03	0.124
Distilled water	1000.00	mL	-	-

The final pH of the medium is 6.9.

3 Details on bacterial genome sequencing results (Table S1)

Table S1 Features of the bacterial genomes sequenced in this study (BioProject PRJEB50809).

**Strain**	***Bosea* sp. 100-5**	***Bosea* sp. 100-5 Mut1**	***Bosea* sp. 3-1B**	***Chelatococcus* sp. 1g-2**	***Chelatococcus* sp. 1g_11**	***Chelatococcus asaccharovorans* WSA4-1**	***Chelatococcus* sp. WSC3-1**	***Chelatococcus asaccharovorans* WSD1-1**	***Chelatococcus* sp. WSG2-a**	***Shinella* sp. WSC3-e**
**Accession number (GCA_)**	930633465	946047605	930633495	930633525	930633505	930633515	930633455	930633485	930633475	945994535
**Genome size (bp)**	5,939,164	5,829,812	5,944,673	7,159,619	7,252,269	7,194,471	6,967,327	7,195,039	7,037,809	7,823,923
**Number of contigs**	4	68	3	6	8	6	5	6	5	12
**GC content (%)**	65.78	65.85	65.78	62.81	62.76	64.14	63.01	64.15	62.98	64.99
**Total number of CDS**	6,101	6,014	6,086	7,265	7,381	7,193	6,914	7,190	7,024	8,023
**Number of tRNA genes**	63	59	63	55	55	51	54	51	55	52
**Number of rRNA genes (5S, 16S, 23S)**	6 (2, 2, 2)	3 (1,1,1)	6 (2, 2, 2)	9 (3, 3, 3)	9 (3, 3, 3)	6 (2, 2, 2)	9 (3, 3, 3)	6 (2, 2, 2)	9 (3, 3, 3)	9 (3, 3, 3)
**Number of miscellaneous RNA genes**	22	22	22	37	39	41	34	40	35	77
**Pseudogenes**	10	6	7	2	2	0	0	0	0	11
**CheckM Completeness (%)**	99.05	98.9	99.05	99.68	99.68	100	99.37	100	99.37	99.87
**CheckM Contamination (%)**	2.92	2.92	2.92	1.35	1.35	1.41	1.61	1.41	1.3	0.58
**Taxonomy (GTDB-tk)**	*Bosea*	*Bosea*	*Bosea*	*Chelatococcus*	*Chelatococcus*	*Chelatococcus asaccharovorans*	*Chelatococcus*	*Chelatococcus asaccharovorans*	*Chelatococcus*	*Shinella*

4 Relative abundance of heterologous proteins in *E. coli* crude extracts (Table S2)

Table S2 Relative protein abundance in crude extracts of *E. coli* Lemo21 (DE3) strains transformed with pET-28(+)-TEV vectors for heterologous expression of BOSEA1005_40015, 40016 or 40030 genes.

Protein	Accession	Gene localization	Length	Mass	Relative abundance (%) after heterologous expression of		
	(NCBI)		(aa)	(kDa)	40015	40016	40030
**heterologous 40015 protein**	n.a.	pET-28(+)-TEV	308	34.7	**0.28**	n.d.	n.d.
**heterologous 40016 protein**	n.a.	pET-28(+)-TEV	486	51.7	n.d.	**0.75**	n.d.
**heterologous 40030 protein**	n.a.	pET-28(+)-TEV	493	52.9	n.d.	n.d.	**0.70**
**LacI**	WP_000805902.1	pET-28(+)-TEV	360	38.6	0.13	0.12	0.20
**KanR**	UQV30771.1	pET-28(+)-TEV	271	31	0.05	0.04	0.04
**elongation factor Tu**	WP_000031784.1	chromosome	394	43.3	10.3	8.3	6.3
**elongation factor G**	WP_000124700.1	chromosome	704	77.5	3.2	2.7	1.4
**chaperonin GroEL**	WP_000729117.1	chromosome	548	57.3	2.4	2.7	4.9
**molecular chaperone DnaK**	WP_000516135.1	chromosome	638	69.1	1.9	1.8	2.6
**glyceraldehyde-3-phosphate dehydrogenase**	WP_000153502.1	chromosome	331	35.5	1.8	2.8	3.3

n.a., not applicable; n.d., not detected

5 NCBI and JGI database search for the BOSEA1005_40015 gene (Table S3)

Table S3 NCBI and JGI metagenome and metatranscriptome datasets that presented the ACE sulfatase gene. The BOSEA1005_40015 sequence was used as query (852 bp, protein with 283 aa). Datasets were obtained from activated sludge samples of various sources as indicated. Numbering as given in Fig. 6.

Number	Source	Accession	Description	Query coordi-nates	Query coverage (%)	Identity (%)	E value	Sampling date	Geographic location
**NCBI database**									
**4**	Metagenome	WBZQ 01256123.1	nitrification reactor of WWTP[a] location 7	1..150	53	99	4E-088	Sept. 2017	Virginia, USA
**4**	Metagenome	WBZQ 01256123.1	nitrification reactor of WWTP[a] location 7	155..283	46	99	2E-073	Sept. 2017	Virginia, USA
**JGI database**									
**2**	Metagenome	3300009873	WWTP, Wenshan plant	1..238	84	100	2E-178	Dec. 2015	Taichung, Taiwan
**3**	Metagenome	3300009870	WWTP, Linkou plant	1..144	51	100	1E-103	Dec. 2015	New Taipei, Taiwan
**6**	Metatranscriptome	3300007323	Klosterneuburg WWTP, MT KNB_C2_LD	1..150	53	99	4E-109	March 2015	Klosterneuburg, Austria
**6**	Metatranscriptome	3300006644	Klosterneuburg WWTP, MT KNB_A2_L	1..109	39	99	1E-75	March 2015	Klosterneuburg, Austria
**6**	Metatranscriptome	3300007595	Klosterneuburg WWTP, MT KNB_T0_6L	177..255	28	100	9E-52	March 2015	Klosterneuburg, Austria
**5**	Metatranscriptome	3300028647	WWTP Weurt	1..283	100	99	0E+00	Aug. 2017	Nijmegen, Netherlands
**1**	Metagenome	3300022303	anaerobic bioreactor, Hunan University, 11	1..283	100	99	0E+00	(2017)[b]	Changsha, China
**1**	Metagenome	3300022241	anaerobic bioreactor, Hunan University, 6	62..283	78	99	1E-164	(2017)[b]	Changsha, China
**1**	Metagenome	3300022302	anaerobic bioreactor, Hunan University, 3	87..283	70	99	1E-143	(2017)[b]	Changsha, China
**1**	Metagenome	3300022302	anaerobic bioreactor, Hunan University, 3	1..66	23	100	8E-40	(2017)[b]	Changsha, China
**1**	Metagenome	3300022215	anaerobic bioreactor, Hunan University, 7	1..152	54	99	3E-110	(2017)[b]	Changsha, China
**1**	Metagenome	3300022238	anaerobic bioreactor, Hunan University, 4	22..144	43	98	1E-86	(2017)[b]	Changsha, China
**1**	Metagenome	3300022291	anaerobic bioreactor, Hunan University, 5	47..166	42	99	4E-85	(2017)[b]	Changsha, China
**1**	Metagenome	3300022291	anaerobic bioreactor, Hunan University, 5	232..283	18	98	1E-26	(2017)[b]	Changsha, China

[a]WWTP – wastewater treatment plant
[b]sampling date not available, database entry created Dec. 2017

6 Detailed description of the LC-MS/MS method for monitoring ANSA

We used an Agilent 1260 Infinity II Series liquid chromatography (LC) system coupled to an AB Sciex QTRAP® 6500+ tandem mass spectrometer (MS/MS) equipped with a Turbo V™ ion source operated with electrospray ionization (ESI) in negative polarity. The enhanced product ion (EPI) spectrum of acetoacetamide-N-sulfonate (ANSA) shown in Fig. 2B was obtained at a scan rate of 10,000 Da s^{-1} using dynamic fill time at a declustering potential of -20 V, an entrance potential of 10 V, a collision energy of -25 V and a collision energy spread of 15 V across the *m/z* range of 55 to 200 for structural elucidation. The ion source-dependent MS parameters, which were kept constant during the whole acquisition, were as follows: curtain gas (CUR): 30 psi; ion spray voltage (IS): -4,500 V; turbo spray temperature (TEM): 450 °C; nebulizer gas (GS1): 60 psi; heater gas (GS2): 60 psi; CAD gas: medium. Nitrogen was used as the curtain and collision gas.

The compound-dependent MS parameters (declustering potential (DP), entrance potential (EP), collision energy (CE) and collision cell exit potential (CXP)) were optimized by direct infusion of the analyte. We used multiple reaction monitoring (MRM) mode combined with the information dependent acquisition (IDA) criteria set at an intensity threshold of 5,000 cps, followed by an EPI as the dependent scan. The MRM transitions and optimized compound-dependent MS parameters for the analyte of interest are listed below.

**Analyte**	**Rt (min)**	**Precursor ion (*m/z*)**	**Product ions (*m/z*)**	**DP (V)**	**EP (V)**	**CE (V)**	**CXP (V)**	**Dwell time (ms)**
ANSA	1.19	179.9	95.8/79.8	-20/-20	-10/-10	-20/-50	-15/-16	40

All data were acquired and processed using Analyst 1.7.2 Software.

7 Preparation of ANSA using chromatographically enriched ACE sulfatase

ANSA was produced with enriched ACE sulfatase activity. Separation of ACE sulfatase from ANSA transforming activity was achieved using size exclusion chromatography (SEC). For that, 200 µL crude extract of ACE-grown *Bosea* sp. 100-5 cells (about 3 µg protein $µL^{-1}$) was loaded onto a Superdex 200 10/300 column (Cytiva, Marlborough, MA, USA) which was equilibrated with 100 mM ammonium bicarbonate buffer (pH 7.82) at 0.5 mL min^{-1} employing a fast protein liquid chromatography (FPLC) system (ÄKTApurifier, Amersham Biosciences Europe, Freiburg, Germany) at room temperature. Fractions were correlated to the molecular weight of proteins by using a protein standard elution curve (thyroglobulin, 670 kDa, γ-globulin, 150 kDa, albumin, 44.3 kDa, ribonuclease A, 13.7 kDa).

In total, 30 fractions with a volume of 0.5 mL were collected and tested for ANSA accumulation at 30 °C in 10 mM Tris-HCl buffer at pH 7.84. For each assay, 2.3 mM ACE and 10% (v/v) of a SEC-fraction were combined and incubated for 1 h. The assay was stopped by adding two volumes of 10 mM malonate buffer (pH 4.0, 60 °C). Substrate consumption and ANSA production was monitored by HPLC. Only two fractions with center points of 14.7 and 15.2 mL (fractions 16 and 17) exhibited significant activity for degrading ACE, amounting to 35 and 30% consumption, respectively. These results indicated that the ACE sulfatase eluted around 70 kDa.

The only product formed from ACE hydrolysis catalyzed by SEC fractions 16 and 17 was ANSA. ANSA was stable under the applied assay conditions and steadily accumulated even upon prolonged incubation of up to two days applying ACE concentrations between 2.3 and 20 mM, indicating that the ANSA hydrolysis activity was in other SEC fractions. For producing sufficient quantities of ANSA, the two SEC fractions showing ACE sulfatase activity were pooled and applied at 50% (v/v) to 20 mM ACE in Tris buffer (10 mM Tris-HCl, pH 7.84). After incubation for two days at 30 °C, ACE was completely transformed. Then, the reaction liquid was filtered through a 3 kDa ultrafilter (Vivaspin; Sartorius, Göttingen, Germany) to obtain a protein-free filtrate. Due to dilution by pre-washing of the filters, ANSA concentrations in the ultrafiltrate typically amounted to about 14 to 16 mM.

8 Calibration factor for HPLC-based ANSA quantification (Fig. S1)

As described in the previous section, in the SEC experiment with crude extract from ACE-grown cells of *Bosea* sp. 100-5, protein fractions were obtained presenting ACE sulfatase activity. Moreover, in the ACE degradation experiments, ANSA seemed to be stable and accumulated over time when monitored by HPLC at 260 nm (photodiode array detector). This is indicative of a stoichiometric conversion of ACE to ANSA.

ACE + H_2O → ANSA

In line with this assumption, the increase in ANSA peak area correlates well with the ACE consumption quantified by HPLC (Fig. S1).

With the ANSA preparation, all SEC fractions were also tested for the amidase activity at 30 °C in the Tris buffer. A substrate concentration of 1.6 mM and 60% (v/v) of each fraction was added. After incubation of 2 h, the assays were stopped by diluting with two volumes of the malonate buffer and substrate consumption was monitored by HPLC. Three fractions with center points of 12.1, 12.6, 13.1 mL (fractions 11, 12, and 13) displayed the highest activity with ANSA consumptions of >90%. Some lower activities were still present in fractions 10 and 14 (between 20 and 30% ANSA consumption), while all other fractions did not show significant activity. This indicates that the ANSA amidase activity was found in fractions representing 150 to 240 kDa. The only product of ANSA hydrolysis (as measured with HPLC) was acetoacetate that was stable under assay conditions.

ANSA + H_2O → Acetoacetate

By combining the datasets obtained from both ACE and ANSA degradation assays, an ANSA calibration factor of 110.6 mAU s μM^{-1} was deduced (Fig. S1A) which can be applied within a concentration range of up to 15 mM (Fig. S1B).

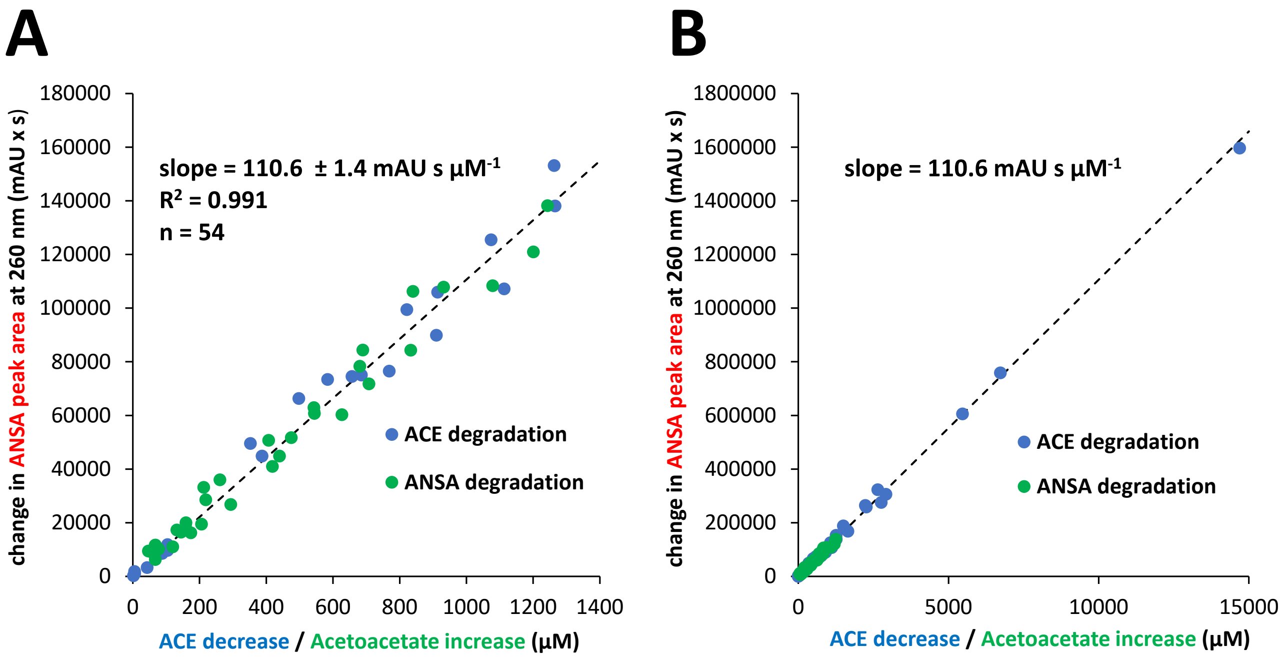

Fig. S1 Change in ANSA peak area at 260 nm when either ACE or ANSA is enzymatically degraded. **(A)** Correlation between ACE decrease (ACE degradation experiment) and acetoacetate formation (ANSA degradation experiment) with the respective change in ANSA peak area recorded by HPLC (photodiode array detector, mAU = milli-absorbance units). For degradation assays, SEC fractions with either ACE- or ANSA-hydrolyzing activities were used. Linear regression analysis results in a calibration factor of 110.6 mAU s μM^{-1} for the quantification of ANSA by HPLC at 260 nm. **(B)** The observed correlation between ANSA peak area and ACE decrease in corresponding degradation experiments applies at least to concentrations of up to 15 mM.

9 Mn^{2+} dependence of *Bosea* sp. 100-5 and MBL-type hydrolase for ACE degradation (Fig. S2)

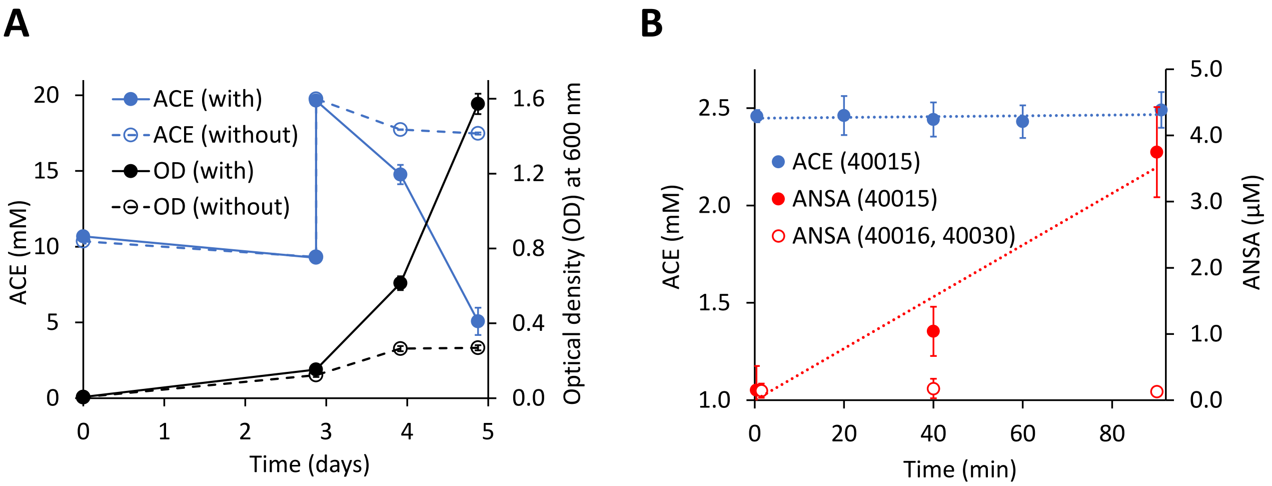

Fig. S2 Mn^{2+} dependence of *Bosea* sp. 100-5 and heterologous MBL-type hydrolase for ACE degradation. **(A)** Fed-batch cultivation of strain *Bosea* sp. 100-5 in DSMZ 462 mineral salt medium with ACE as sole carbon and energy source. The concentration of Mn^{2+} in the medium amounts to 0.15 µM (see recipe for DSMZ 462 medium provided above in section 2). This allowed only very limited growth and ACE degradation monitored as optical density (OD) at 600 nm and by HPLC, respectively (open symbols, "without"). In contrast, growth and degradation were substantially stimulated by supplementation with 3.7 µM $MnSO_4$ (closed symbols, "with"). Cultures were incubated at 30°C. Values shown refer to mean and SD of at least five independent experiments. **(B)** ACE sulfatase activity assay with heterologous BOSEA1005_40015 not supplemented with Mn^{2+}. Only very low ACE sulfatase activities were achieved with crude extracts containing heterologous BOSEA1005_40015 enzyme (closed symbols, 1330 µg mL^{-1} total protein in assay), which were obtained from *E. coli* Lemo21 (DE3) expressing the hydrolase gene in lysogeny broth medium without supplements (despite antibiotics and inducer). In addition, assay buffer was not supplemented with $MnCl_2$. In stark contrast, conversion of ACE to ANSA in BOSEA1005_40015 crude extracts supplemented with $MnCl_2$ (0.5 mM during expression and 1 mM in assay buffer) is about 300 times higher (see Fig. 4A). In comparison, ANSA formation from ACE in crude extracts containing heterologous amidases (either BOSEA1005_40016 or 40030, open symbols, 1330 µg mL^{-1} total protein in assay, see also Fig. 4A) is insignificant (below 0.2 µM monitored by LC-MS/MS). Values given represent mean and SD of at least four independent experiments.

10 Characterization of mutant strain *Bosea* sp. 100-5 Mut1 (Figs. S3 and S4)

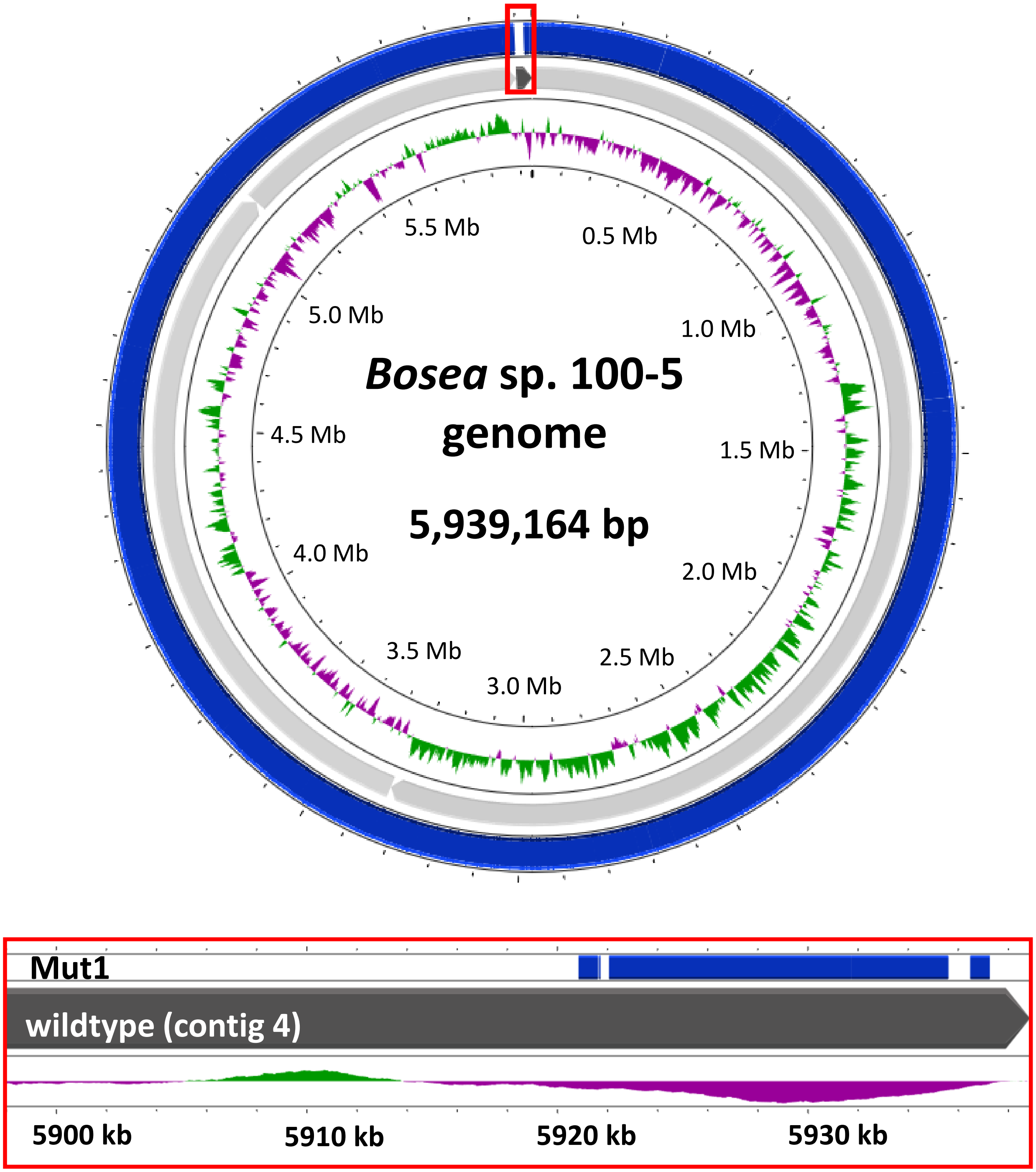

Fig. S3 Alignment of the *Bosea* sp. 100-5 Mut1 genome (blue) against the *Bosea* sp. 100-5 genome (grey). The 41-kb zoom (red framed box) shows the gaps found in the Mut1 genome, amounting to a total deletion of 26.3 kb. The figure was generated with Proksee.

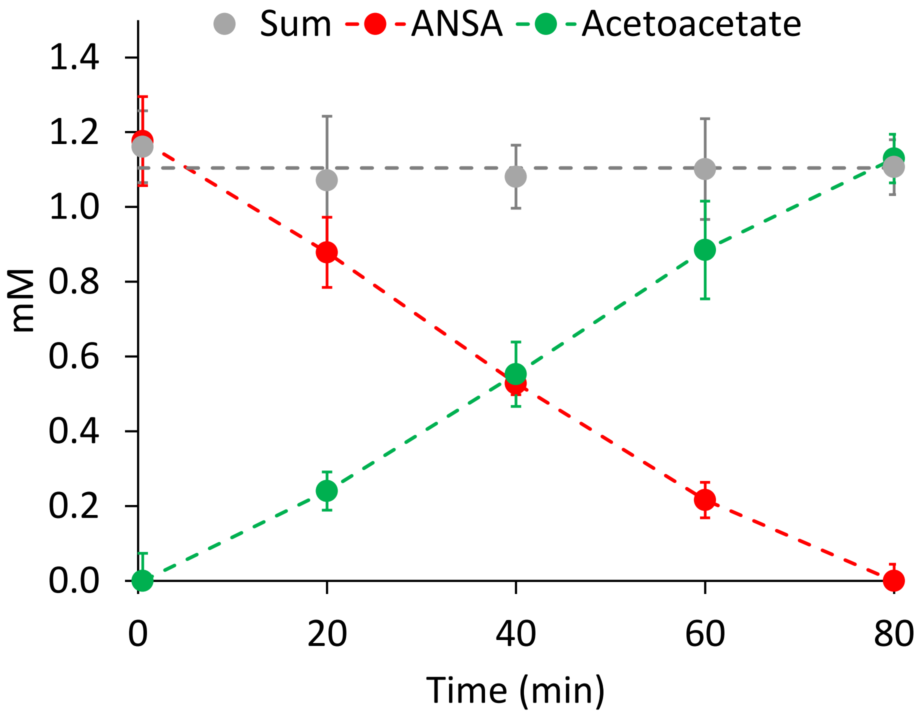

Fig. S4 ANSA degradation assay with crude extracts obtained from *Bosea* sp. 100-5 Mut1 cells. The strain was grown in DSMZ 461 (recipe described in section 1) supplemented with 20 mM 3-hydroxybutyrate (used as carbon and energy source) and 2.5 mM ACE (not degraded during cultivation). At 30 °C and within 80 min, about 1.2 mM ANSA was completely converted to acetoacetate by the crude extracts (260 µg mL^{-1} total protein). Values given represent mean and SD of at least five independent experiments.

11 Functional assignment for predicted proteins encoded in ACE cluster 2 (Fig. S5)

CHELA1g11_60039: 480 aa protein, permease system that can likely operate as a chemiosmotic transporter for the uptake of small anions. Distantly related to a putative short-chain fatty acid transporter found in *E. coli* (UniProt ID P76460; 34% identity at 94% query coverage). In *Bosea* sp. 100-5 and *Bosea* sp. 3-1B, the homologous gene is disrupted/incomplete and not part of the gene cluster (Fig. 3). See also Fig. S7 for comparison of gene clusters.

CHELA1g11_60040 / BOSEA31B_30045: Another TauE/SafE export system (see ACE cluster 1, BOSEA1005_40018) involved in excretion of small anions. The CHELA1g11_60040 / BOSEA31B_30045 protein shows only low similarity to BOSEA1005_40018 (44% identity at 10% query coverage, blastp). See also Fig. S7 for comparison of gene clusters.

BOSEA1005_40029: The gene product (311 aa) is predicted to be a helix-turn-helix domain bearing transcriptional regulator of the LysR family. In the UniProt database, one of the closest matches (30% identity 78% query coverage) is AlsR from *Bacillus subtilis* 168 (ID Q04778), which is involved in regulating the expression of acetoin synthesis genes (Renna et al., 1993). Due to the highly conserved co-occurrence of BOSEA1005_40029 and BOSEA1005_40030 sequences in ACE cluster 2 found in ACE-degrading strains, the LysR family protein might be involved in the regulation of the expression of the ANSA amidase gene.

BOSEA1005_40030: “amidase 2”, ANSA amidase bearing the amidase signature sequence. The total protein possesses 472 aa and a molecular weight of 50.5 kDa. Residues of the catalytic Ser Ser Lys triad for attacking amide groups are K83, S158 and S182. The closest match of a characterized enzyme is an amidase from *Rhodococcus erythropolis* TA37 (UniProt ID K9NBS6, Fig. S5) showing 40% identity at 95% query coverage. This amidase from strain TA37 is specific for short-chain N-substituted amides (Lavrov et al., 2010). Likewise, BOSEA1005_40030 is only distantly related to BOSEA1005_40016 (“amidase 1”) of the sulfatase gene cluster (sharing only 39% identity residues at 90% query coverage). As discussed in the manuscript, a sequence very similar to BOSEA1005_40030 can be found in *Paraburkholderia sartisoli* LMG 24000 (Fig. S5). See also Fig. S7 for comparison of gene clusters. However, the latter strain does not contain the ACE sulfatase (BOSEA1005_40015) and cannot degrade ACE (as tested in this study).

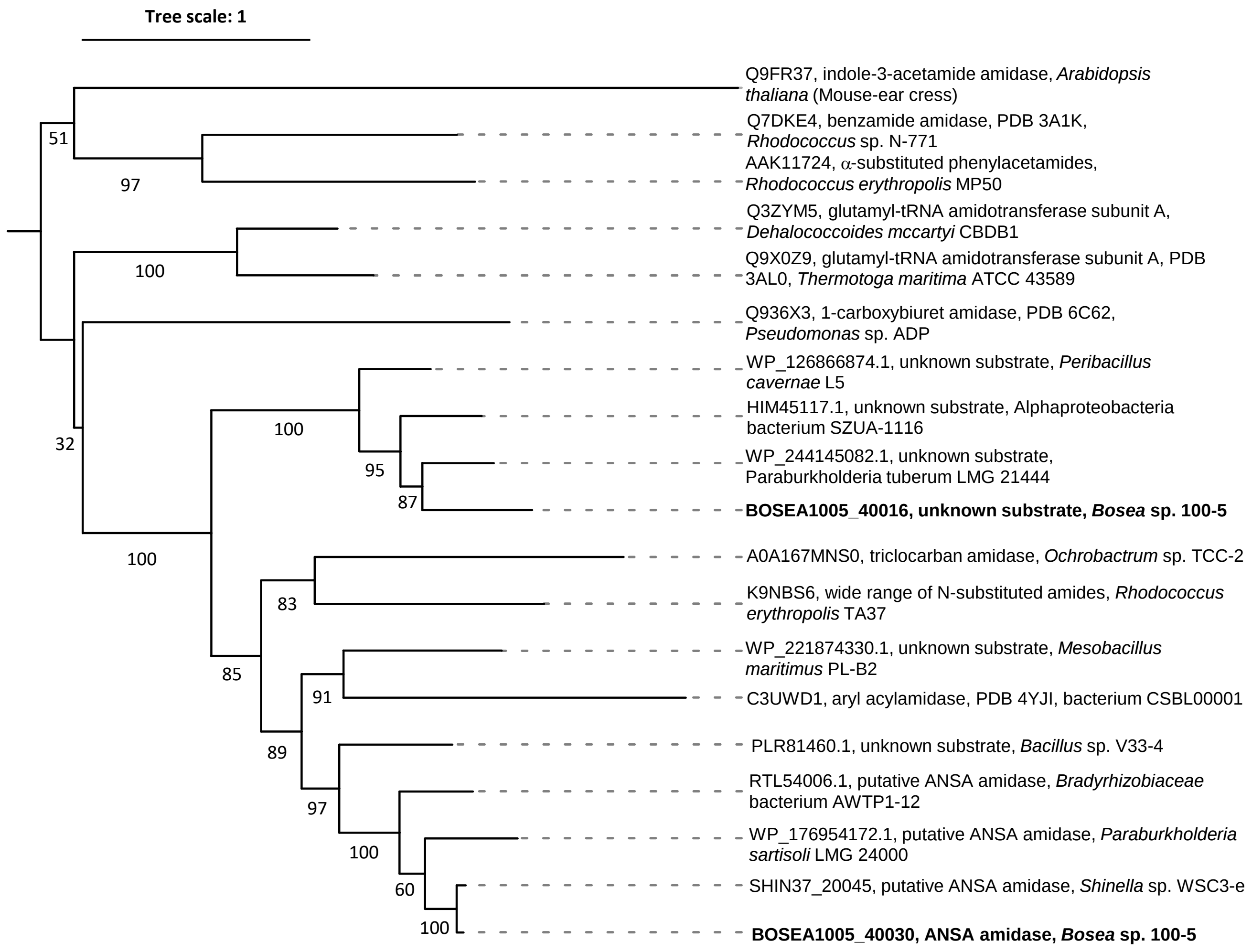

Fig. S5 Maximum-likelihood tree of 19 amidase sequences with the best fitting evolution model (Kalyaanamoorthy et al., 2017). Tree was midpoint-rooted, and ultrafast bootstrap support values (%) were calculated from 1000 replicates (Hoang et al., 2017). BOSEA1005_40016 and 40030 amidases from *Bosea* sp. 100-5 (highlighted in bold) are found in different tree branches. The alignment of the 19 amidases were performed in MEGA11 using ClustalW with default parameters (Tamura et al., 2021). IQ-TREE was used to infer the substitution model and build the Maximum-likelihood tree (Nguyen et al., 2014), and the tree was visualized and modified in i-TOL (Letunic and Bork, 2021).

12 Map and annotations of the ACE plasmid found in strain *Chelatococcus* sp. 1g-11 (Fig. S6)

Fig. S6 Map and annotations of the ACE plasmid found in strain *Chelatococcus* sp. 1g-11. Color code as used in Fig. 5. The plasmid figure was generated with Proksee.

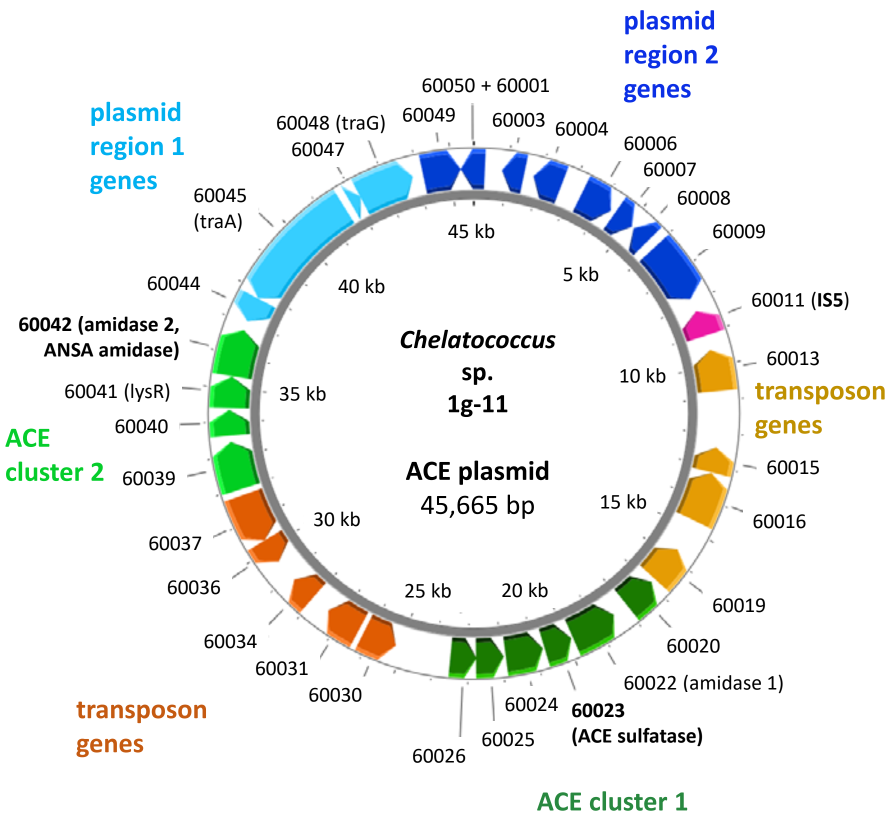

Predicted functions (locus tag prefix CHELA1G11_)

Locus tag	Gene length (bp)	Predicted function of gene product	Plasmid region
60001+50	720[a]	Protein of unknown function	Plasmid region 2
60003	672	Chromosome partitioning protein	Plasmid region 2
60004	876	Replication protein A	Plasmid region 2
60006	1008	Conserved protein of unknown function	Plasmid region 2
60007	651	Homocitrate synthase	Plasmid region 2
60008	567	Serine recombinase PinE	Plasmid region 2
60009	1875	Conserved protein of unknown function	Plasmid region 2
60011	783	Transposase	IS5 family element
60013	1239	Integrase/recombinase	Transposon
60015	756	Insertion sequence ATP-binding protein	Transposon
60016	1512	Transposase	Transposon
60019	1110	Transposase	Transposon
60020	1008	TauE-like small molecular weight anion export system	ACE cluster 1
60022	1398	Amidase 1, unknown substrate	ACE cluster 1
60023	852	MBL-type hydrolase, ACE sulfatase	ACE cluster 1
60024	1134	ABC type import system, substrate-binding subunit	ACE cluster 1
60025	834	ABC type import system, transmembrane subunit	ACE cluster 1
60026	786	ABC type import system, ATP-binding subunit	ACE cluster 1
60030	1032	Transposase	Transposon
60031	1083	Transposase	Transposon
60034	777	Putative RNA-directed DNA polymerase	Transposon
60036	759	AAA family ATPase	Transposon
60037	1497	Transposase	Transposon
60039	1443	Low molecular weight anion importer	ACE cluster 2
60040	765	TauE/SafE system, low molecular weight anion export	ACE cluster 2
60041	936	LysR-like transcriptional regulator	ACE cluster 2
60042	1419	Amidase 2, ANSA amidase	ACE cluster 2
60044	747	Family of unknown function (DUF6118)	Plasmid region 1
60045	3777	Conjugal transfer protein TraA	Plasmid region 1
60047	327	Mobilization protein, MobC-like	Plasmid region 1
60048	1680	Type IV secretion system protein TraG	Plasmid region 1

[a]complete CDS after manually circularizing the contig, identical to the homologous CDS of MAG ACE_PRO6 (MBX3540288.1)

13 Comparison of gene clusters bearing the gene for the ANSA amidase (Fig. S7)

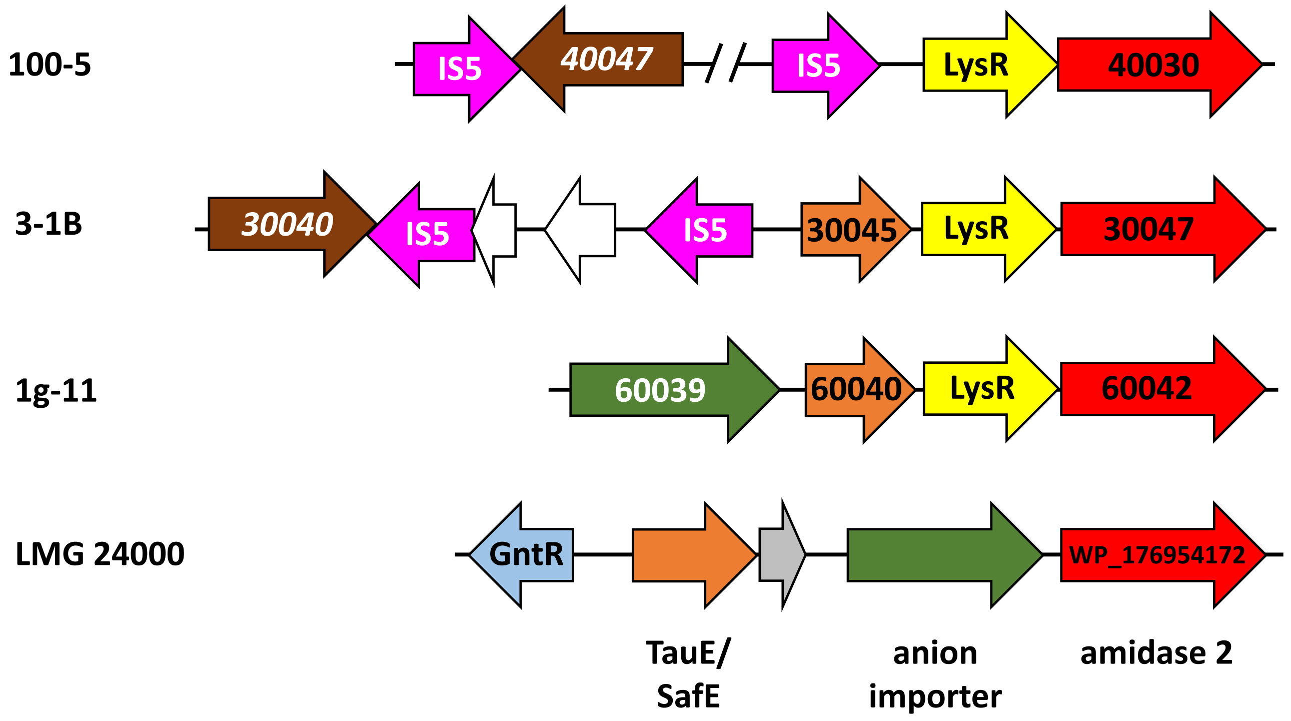

Fig. S7 Comparison of gene clusters bearing the gene for the ANSA amidase ("amidase 2"). In *Bosea* sp. 100-5, *Bosea* sp. 3-1B and *Chelatococcus* sp. 1g-11, the cluster is found on the ACE plasmid, whereas it is located in *Paraburkholderia sartisoli* LMG 24000 on a contig of 454,572 bp (NZ_FNRQ01000004.1) that is likely part of the chromosome. Sequences of the amidase, the TauE/SafE exporter (absent in *Bosea* sp. 100-5) and the LysR-like transcriptional regulator are identical in the ACE degraders. The anion importer is only complete in strain 1g-11 (480 aa). In the *Bosea* strains, it can be interpreted as a pseudogene (BOSEA1005_40047, BOSEA31B_30040) located outside the gene cluster and resulting in a protein with missing N- and C-termini. The CDS start has been disrupted by the IS5 family transposase (Fig. 3). In total, only about 330 aa can still be aligned to CHELA1g11_60039 and related transporters. In contrast, the importer is conserved in strain LMG 24000 (sharing 75% protein sequence identity at 93% coverage with CHELA1g11_60039). Likewise, the amidase of this strain is closely related to the ANSA amidase (66% identity at 99% coverage). The TauE/SafE system, however, does not show significant match (blastp) on sequence level to the protein encoded in the amidase gene cluster of the ACE degraders.

14 Possible two-step enzymatic hydrolysis of ACE-related chemicals (Fig. S8)

Among chemicals structurally related to ACE are, e.g., the anticonvulsant and antiepileptic drug topiramate (Privitera, 1997) and antiviral nucleotide sulfamates (Winum et al., 2005). A hydrolytic attack at the sulfur atom of these O-substituted sulfamates may release sulfamic acid/the sulfamate anion. However, regarding the subsequent amidase reaction, also an N-derivatization of the parental compound with a carbonyl carbon would be a prerequisite for the evolution of a two-step hydrolase pathway for ACE degradation. Furthermore, sulfamic acid derivatives used as pharmaceuticals can only cause micropollutions in wastewater, thus, being insignificant growth substrates as well. Rather, organic sulfamates directly affecting bacterial growth could explain the evolution of the ACE gene clusters.

In this context, ascamycin and other natural or synthetic aminoacyl sulfamate ribonulceosides acting as inhibitors of aminoacyl-tRNA synthetases might play a role. Although enzymatic hydrolysis in *Xanthomonas campestris* seems to affect initially the amide and is reported to be catalyzed by a proline iminopeptidase (UniProt ID P52279) not related to amidase signature sequence hydrolases (Sudo et al., 1996), the combination of a BOSEA1005_40015-like sulfatase with BOSEA1005_40016- or 40030-like amidases in other bacteria might neutralize the antibiotic activity and release sulfamic acid/sulfamate as one reaction product (Fig. S8A).

Likewise, sulfonamide antibiotics might be relevant, as these compounds have already been applied for many decades (Henry, 1943; Seydel, 1968) and resistance against them can be strongly selective. As shown in Fig. S8B, depending on the sulfonamide structure, a sulfatase-catalyzed attack might lead to formation of an amide-N-sulfonate intermediate that could be further hydrolyzed by an amidase to the corresponding carboxylic and sulfamic acid ions.

Biochemical characterization of the BOSEA1005_40015, 40016 and 40030 enzymes by elucidating structures and substrate specificity may be worthwhile and could extend our knowledge on sulfamate and sulfonamide degradation mechanisms. Currently, however, the origin of the individual ACE gene clusters and the evolutionary drivers that established the ACE pathway specifically in alphaproteobacterial genera remains elusive.

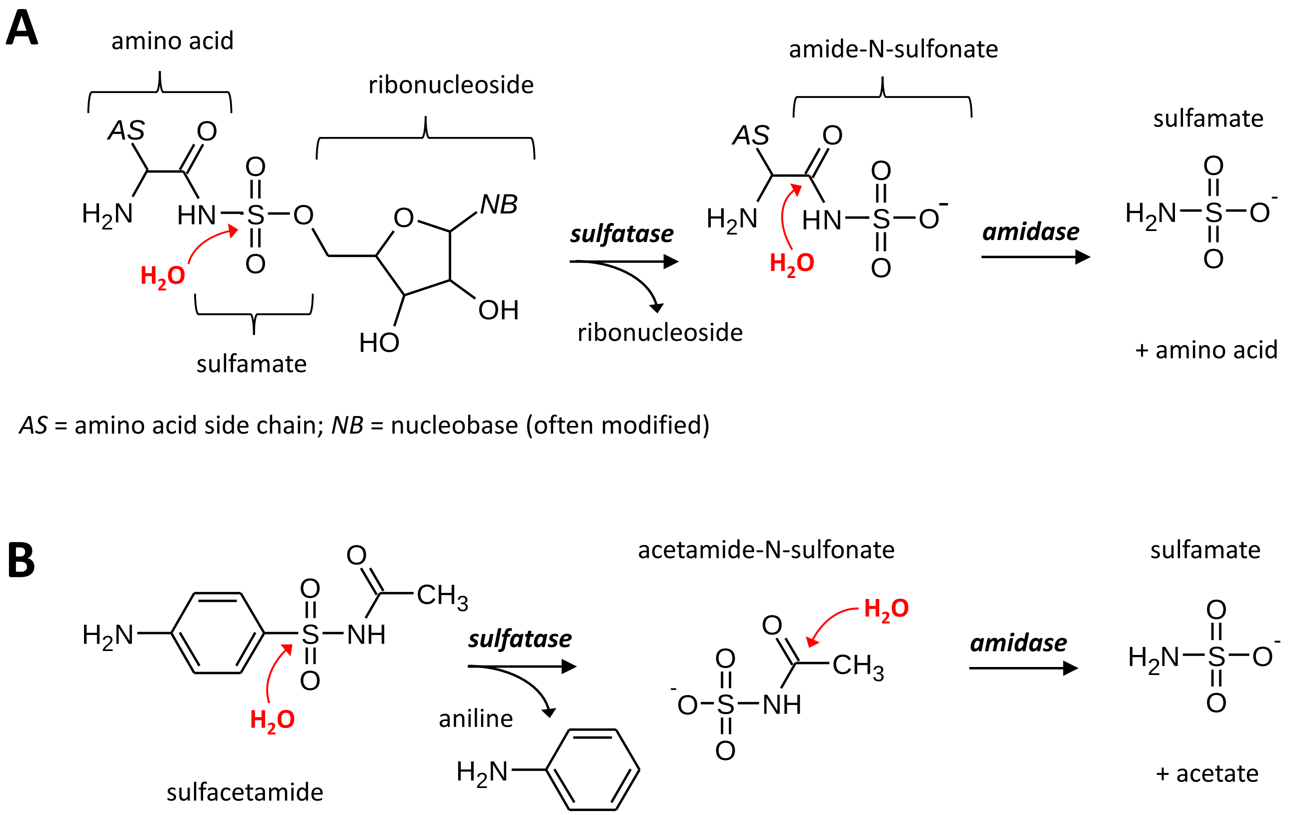

Fig. S8 Hypothetical pathways for the two-step hydrolysis of ACE-related chemicals catalyzed by BOSEA40015_40015-like sulfatases and BOSEA1005_40016- or 40030-like amidases. **(A)** Hydrolysis of aminoacyl sulfamate ribonucleotide antibiotics could proceed via an amide-N-sulfonate as intermediate and could form the sulfamate ion as end product. **(B)** Likewise, the sulfonamide antibiotic sulfacetamide might be degraded via acetamide-N-sulfonate.

15 Blastn results with ACE cluster 1 and 2 sequences as query against metagenome assembly GCA_009612895.1 and corresponding SRA datasets (Figs. S9 and S10)

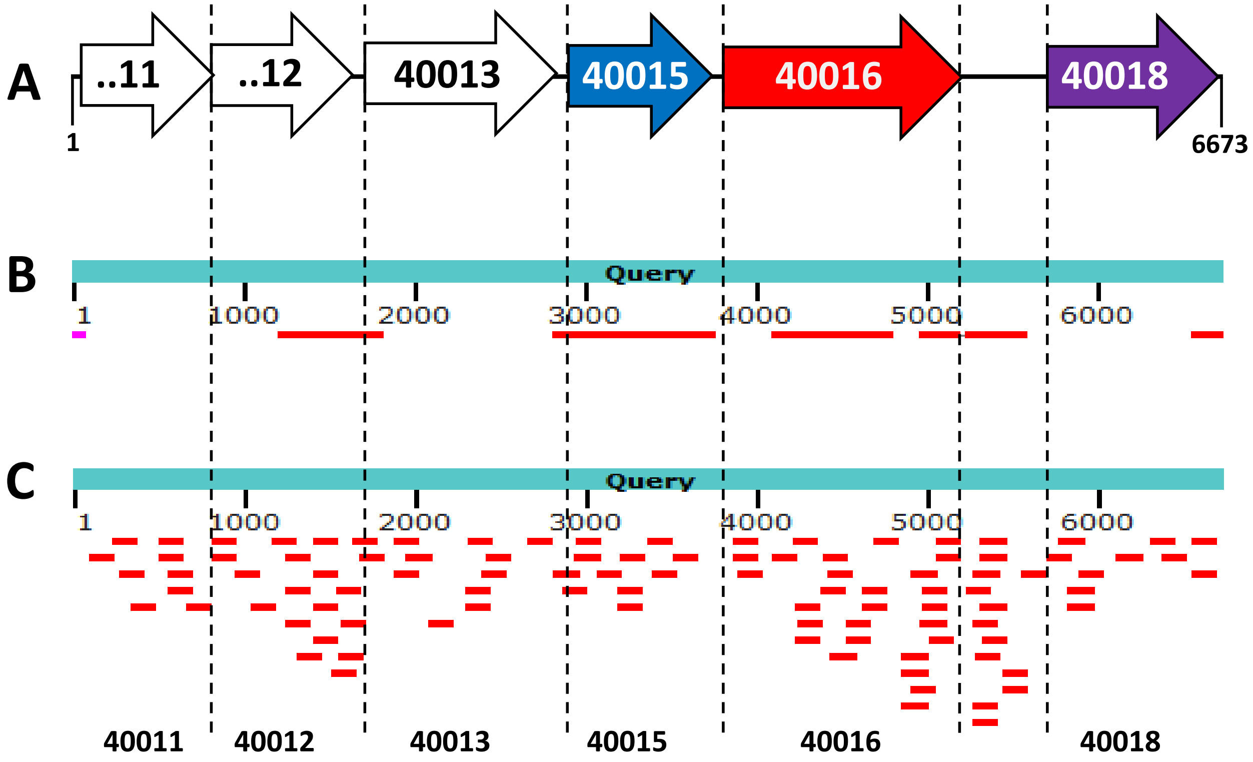

Fig. S9 Blastn results with ACE cluster 1 sequence as query against metagenome assembly GCA_009612895.1 and corresponding SRA datasets. **(A)** Genes of ACE cluster 1 from strain *Bosea* sp. 100-5, prefix BOSEA1005_. **(B)** Search against the assembly GCA_009612895.1 (WGS project WBZQ: Activated sludge metagenome isolate JAMM) that based on SRA datasets SRX6584288 and SRX6584283 resulting from samples SAMN12344353 and SAMN12344354, respectively, which were taken from activated sludge of a nitrification reactor of a wastewater treatment plant ("location 7", Virginia, USA, September 2017). For the alignment with ACE cluster 1, only sequences showing 98 to 100% identity to the query sequence were considered. **(C)** Search results directly using the SRA datasets SRX6584288 and SRX6584283. For the alignment with ACE cluster 1, only sequences showing 99 to 100% identity to the query sequence were considered.

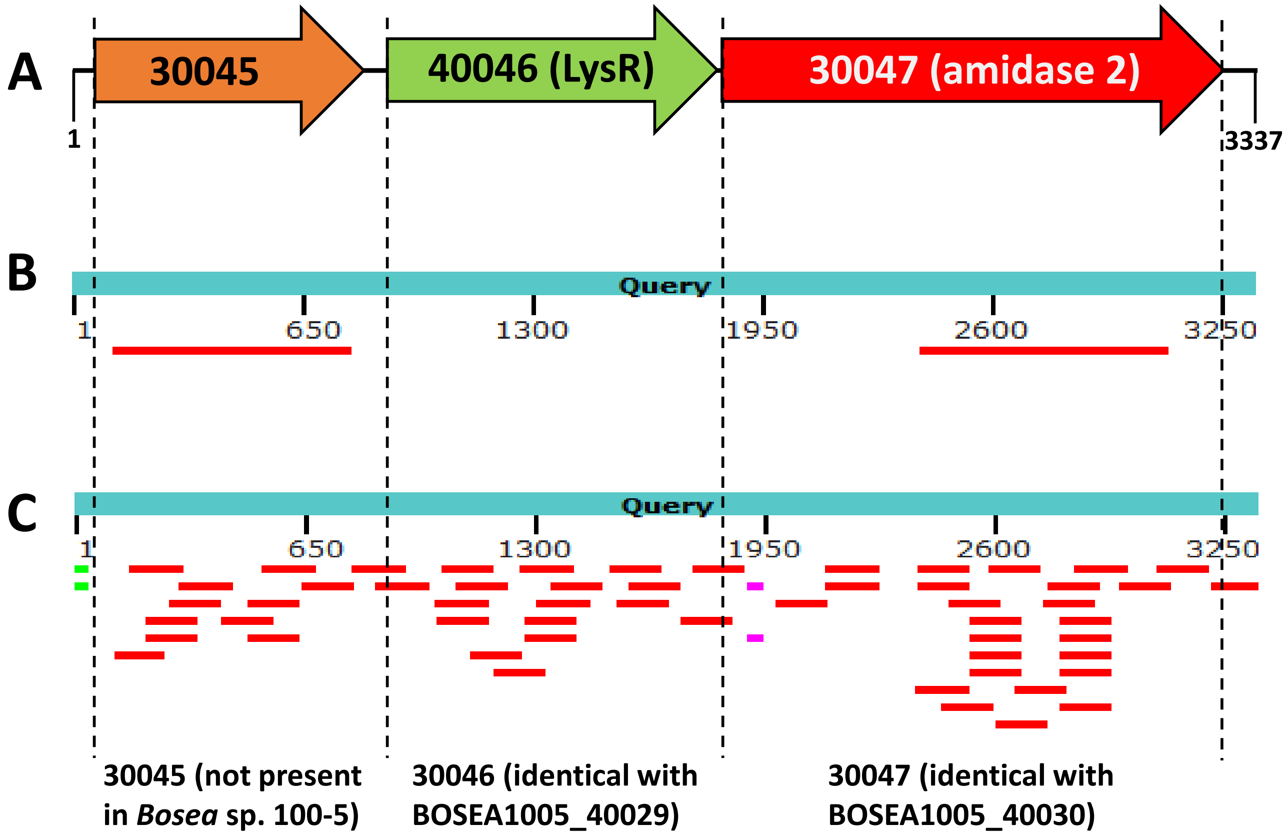

Fig. S10 Blastn results with ACE cluster 2 sequence as query against metagenome assembly GCA_009612895.1 and corresponding SRA datasets. **(A)** Genes of ACE cluster 2 from strain *Bosea* sp. 3-1B, prefix BOSEA31B_. **(B)** Search against the assembly GCA_009612895.1 (WGS project WBZQ: Activated sludge metagenome isolate JAMM) that based on SRA datasets SRX6584288 and SRX6584283 resulting from samples SAMN12344353 and SAMN12344354, respectively, which were taken from activated sludge of a nitrification reactor of a wastewater treatment plant ("location 7", Virginia, USA, September 2017). For the alignment with ACE cluster 2, only sequences showing 98 to 100% identity to the query sequence were considered. **(C)** Search results directly using the SRA datasets SRX6584288 and SRX6584283. For the alignment with ACE cluster 2, only sequences showing 99 to 100% identity to the query sequence were considered.

16 **Blastn results with ACE cluster 1 and 2 sequences as query against SRA datasets of BioProject PRJNA904380 (Figs. S11)**

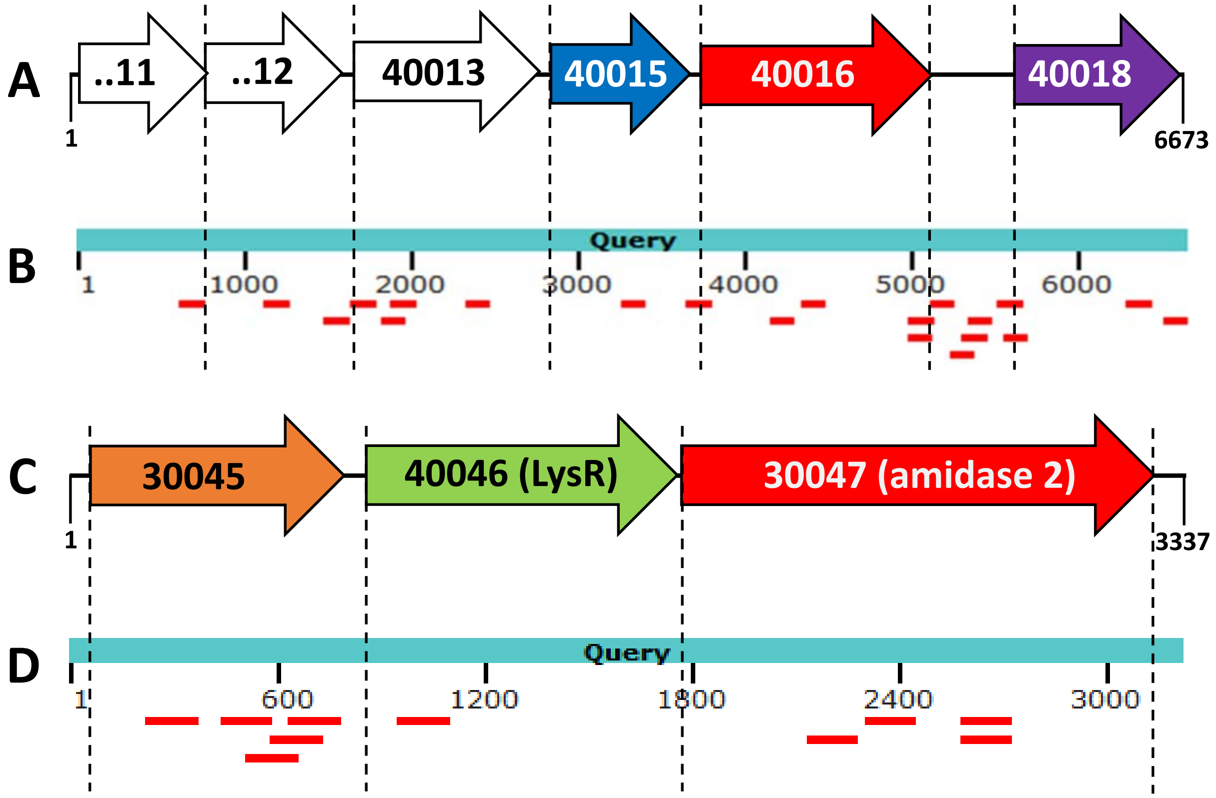

Fig. S11 Blastn results with ACE cluster 1 and 2 sequences as query against selected SRA datasets (SRX18713065, SRX18713066, SRX18713067, SRX18713072, SRX18713073, SRX18713074) of BioProject PRJNA904380 resulting from pond sediment samples collected at the WWTP of Christchurch, New Zealand, in December 2019. **(A)** Genes of ACE cluster 1 from strain *Bosea* sp. 100-5, prefix BOSEA1005_. **(B)** Search results using the SRA datasets. For the alignment with ACE cluster 1, sequences showing 97 to 100% identity to the query sequence were considered. **(C)** Genes of ACE cluster 2 from strain *Bosea* sp. 3-1B, prefix BOSEA31B_. **(D)** Search results using the SRA datasets. For the alignment with ACE cluster 2, sequences showing 97 to 100% identity to the query sequence were considered.

17 References

Henry, R.J. (1943). THE MODE OF ACTION OF SULFONAMIDES. *Bacteriol Rev* 7(4)**,** 175-262. doi: 10.1128/br.7.4.175-262.1943.

Hoang, D.T., Chernomor, O., von Haeseler, A., Minh, B.Q., and Vinh, L.S. (2017). UFBoot2: Improving the Ultrafast Bootstrap Approximation. *Molecular Biology and Evolution* 35(2)**,** 518-522. doi: 10.1093/molbev/msx281.

Kalyaanamoorthy, S., Minh, B.Q., Wong, T.K.F., von Haeseler, A., and Jermiin, L.S. (2017). ModelFinder: fast model selection for accurate phylogenetic estimates. *Nature Methods* 14(6)**,** 587-589. doi: 10.1038/nmeth.4285.

Lavrov, K.V., Zalunin, I.A., Kotlova, E.K., and Yanenko, A.S. (2010). A new acylamidase from Rhodococcus erythropolis TA37 can hydrolyze N-substituted amides. *Biochemistry (Moscow)* 75(8)**,** 1006-1013. doi: 10.1134/S0006297910080080.

Letunic, I., and Bork, P. (2021). Interactive Tree Of Life (iTOL) v5: an online tool for phylogenetic tree display and annotation. *Nucleic Acids Research* 49(W1)**,** W293-W296. doi: 10.1093/nar/gkab301.

Nguyen, L.-T., Schmidt, H.A., von Haeseler, A., and Minh, B.Q. (2014). IQ-TREE: A Fast and Effective Stochastic Algorithm for Estimating Maximum-Likelihood Phylogenies. *Molecular Biology and Evolution* 32(1)**,** 268-274. doi: 10.1093/molbev/msu300.

Privitera, M.D. (1997). Topiramate: a new antiepileptic drug. *Ann Pharmacother* 31(10)**,** 1164-1173. doi: 10.1177/106002809703101010.

Renna, M.C., Najimudin, N., Winik, L.R., and Zahler, S.A. (1993). Regulation of the Bacillus subtilis alsS, alsD, and alsR genes involved in post-exponential-phase production of acetoin. *J Bacteriol* 175(12)**,** 3863-3875. doi: 10.1128/jb.175.12.3863-3875.1993.

Seydel, J.K. (1968). Sulfonamides, Structure-Activity Relationship, and Mode of Action: Structural Problems of the Antibacterial Action of 4-Aminobenzoic Acid (PABA) Antagonists. *Journal of Pharmaceutical Sciences* 57(9)**,** 1455-1478. doi: https://doi.org/10.1002/jps.2600570902.

Sudo, T., Shinohara, K., Dohmae, N., Takio, K., Usami, R., Horikoshi, K., et al. (1996). Isolation and characterization of the gene encoding an aminopeptidase involved in the selective toxicity of ascamycin toward Xanthomonas campestris pv. citri. *Biochem J* 319 (Pt 1)(Pt 1)**,** 99-102. doi: 10.1042/bj3190099.

Tamura, K., Stecher, G., and Kumar, S. (2021). MEGA11: Molecular Evolutionary Genetics Analysis Version 11. *Molecular Biology and Evolution* 38(7)**,** 3022-3027. doi: 10.1093/molbev/msab120.

Winum, J.Y., Scozzafava, A., Montero, J.L., and Supuran, C.T. (2005). Sulfamates and their therapeutic potential. *Med Res Rev* 25(2)**,** 186-228. doi: 10.1002/med.20021.
